# Molecular diversity and evolutionary trends of cysteine-rich peptides from the venom glands of Chinese spider *Heteropoda venatoria*

**DOI:** 10.1038/s41598-021-82668-5

**Published:** 2021-02-05

**Authors:** Jie Luo, Yiying Ding, Zhihao Peng, Kezhi Chen, Xuewen Zhang, Tiaoyi Xiao, Jinjun Chen

**Affiliations:** 1 College of Bioscience and Biotechnology, Hunan Agricultural University, Changsha, 410128 People’s Republic of China; 2 College of Animal Science and Technology, Hunan Agricultural University, Changsha, 410128 People’s Republic of China; 3 Hunan Provincial Engineering Technology Research Center for Cell Mechanics and Function Analysis, Changsha, 410128 People’s Republic of China

**Keywords:** Biochemistry, Evolution, Molecular biology, Zoology

## Abstract

*Heteropoda venatoria* in the family Sparassidae is highly valued in pantropical countries because the species feed on domestic insect pests. Unlike most other species of Araneomorphae, *H. venatoria* uses the great speed and strong chelicerae (mouthparts) with toxin glands to capture the insects instead of its web. Therefore, *H. venatoria* provides unique opportunities for venom evolution research. The venom of *H. venatoria* was explored by matrix-assisted laser desorption/ionization tandem time-of-flight and analyzing expressed sequence tags. The 154 sequences coding cysteine-rich peptides (CRPs) revealed 24 families based on the phylogenetic analyses of precursors and cysteine frameworks in the putative mature regions. Intriguingly, four kinds of motifs are first described in spider venom. Furthermore, combining the diverse CRPs of *H. venatoria* with previous spider venom peptidomics data, the structures of precursors and the patterns of cysteine frameworks were analyzed. This work revealed the dynamic evolutionary trends of venom CRPs in *H. venatoria*: the precursor has evolved an extended mature peptide with more cysteines, and a diminished or even vanished propeptides between the signal and mature peptides; and the CRPs evolved by multiple duplications of an ancestral ICK gene as well as recruitments of non-toxin genes.

## Introduction

Spiders (*Araneae*), including 48,504 described species grouped into 4156 genera and 120 families https://wsc.nmbe.ch/ (December 2019), have high biological and ecological diversity. Most of spiders employ chemical and pharmacological complicated venoms to subdue their prey rapidly. Spider venoms contain rich compounds, including proteins, peptides, and low-molecular-weight components. The predominant peptides of spider venoms are secretory cysteine-rich peptides (CRPs) with multiple disulfide bonds that provide stability and resistance to protease degradation^1^. There has been much interest in studying the biochemical and structural properties^2,3^, pharmacological applications^4^, evolution and diversification of secretory cysteine-rich peptide toxin^5–9^. Up to now, the venoms of snakes, scorpions, and *Conus snails* are much more deeply understood than those of spiders partly since spiders have considerable species and complicated venoms^10,11^.

The huntsman spider (*Heteropoda venatoria* Carl Linnaeus, 1767) is a member of family Sparassidae, a common name giant crab spider, and takes more than 250 days to complete their life cycle, which leads to the collection of natural venoms is limited^12^. Adult specimens have a flattened body length of about 25 mm with eight long, slightly hairy legs spanning 60 to 120 mm. A yellow to cream clypeus just in front of the eyes is one of the main features of *H. venatoria.* The female has a larger abdomen with an overall brown body. Usually, an egg sac up to about 25 mm wide was carried with her pedipalps under its body. The male has a slender body and long legs, a distinctive pattern on his carapace. This species is found in many tropical and subtropical regions globally and can’t survive outside during sub-freezing temperatures. In the present study, the spiders were found from basements, barns, and greenhouses of our scientific research farm in summer. The huntsman spiders do not spin webs. They are known to hunt and feed on living insects with their exceptional agility and speed at night. They can stay and run on a smooth vertical surface and contort and squeeze their large body to fit into surprisingly small cracks and crevices, which give them a strong advantage both in predation and evading predators. Almost as soon as they catch their prey, the spiders paralyze them by injecting with the venoms, from glands extending from the chelicerae into the cephalothorax. The spider is considered a beneficial resident of households because it can hunt pests efficiently and does no harm to people^13^. The venom of *H. venatoria* contains hundreds of peptides with severe toxicities on *Periplaneta americana* (LD_50_: 28.18 mg/g of body weight), and venom neurotoxins target specific types of insect ion channels and receptors, which have broadly applied potential as insecticides in pest control^14^. Many studies have focused on the activity and composition of the venom of *H. venatoria*. However, the diversity of CRPs in the venom, and the evolutionary relationship in the phylogenetic framework has not been explored.

**Figure 1:**
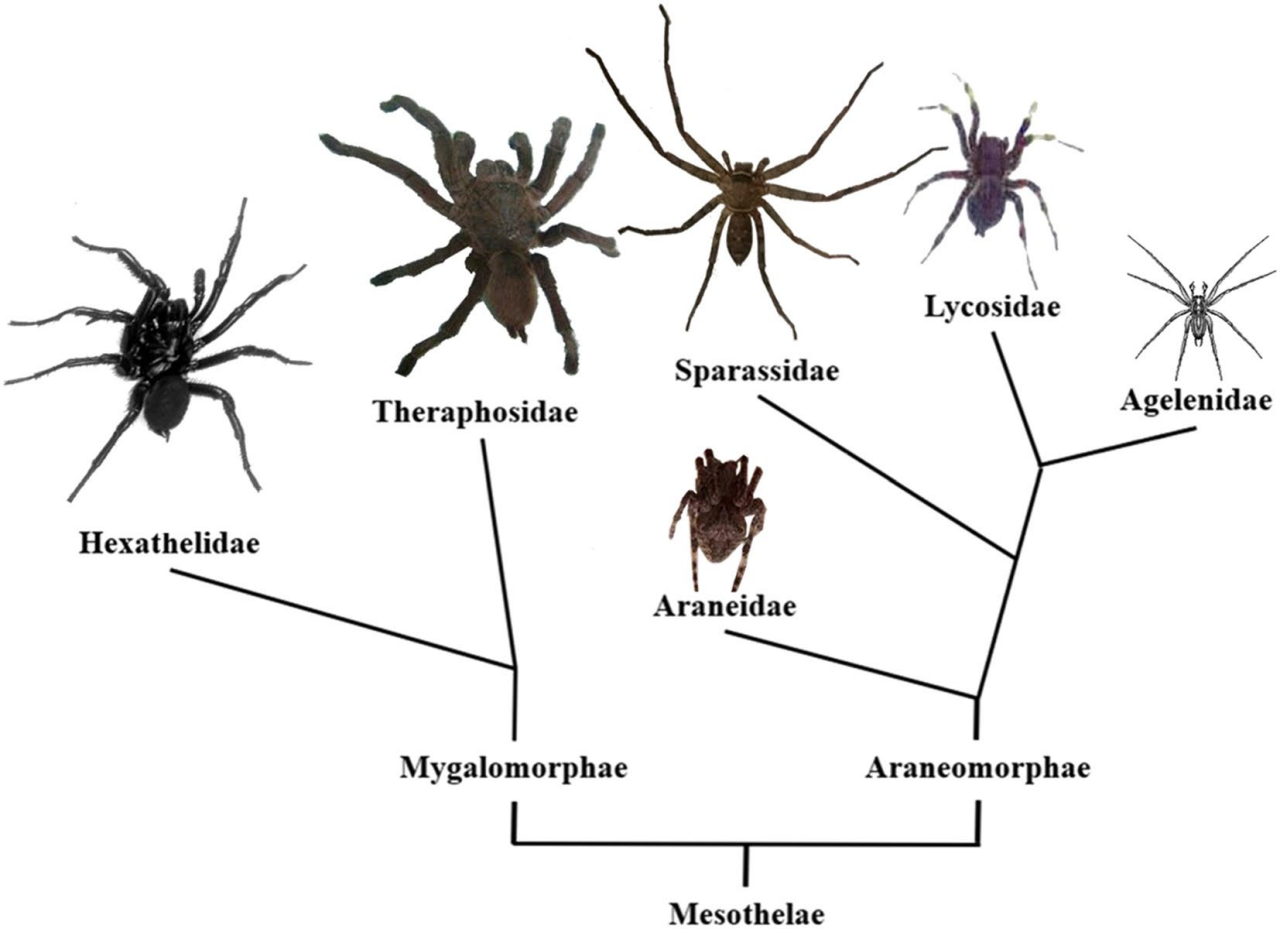
Simplified phylogenetic tree of spider families with the physique in the relative size of the representative species of each family. Modified from Kuhn-Nentwig et al. (2011)^11^. *Hadronyche versuta*^15^ in Hexathelidae, *Chilobraechys guangxiensis* in Theraphosidae, *Araneus trifolium* in Araneidae, *Heteropoda venatoria* in Sparassidae, *Lycosa singoriensis*^16^ in Lycosidae, and *Agelenopsis potteri*^17^ in Agelenidae are mentioned in the present work.

The phylogenetic relationships amongst the spider families whose venom CRPs have been well described show *H. venatoria* (in Sparassidae) locate between Theraphosidae and Lycosidae (Fig. 1). Comparing Mygalomorphae species (*Cyriopagopus schmidti*, *Cyriopagopus hainanus*, *Grammostola rosea*, *Chilobrachys guangxiensis*) to Araneomorphae species (*Lycosa singoriensis*, *Dolomedes mizhoanus*, *Araneus ventricosus*), the body size of *H. venatoria* is medium between the two suborders, and its family, Sparassidae, belongs to the more primitive Araneomorphae^11^. However, *H. venatoria* subdues prey by long legs, strong chelicerae, and complex venom, which seems similar to tarantula’s predation behavior. Unlike web-forming spiders (*A. ventricosus, A. orientalis *et al*.*) that weave cobwebs before prey and then eat after wrapping its prey with cobwebs.

Based on the comparisons of morphology and predation habits of *H. venatoria* along with other spiders, as well as its phylogenetic classification, we believe that the investigation of the genetic coding products in its venom can contribute to the understanding of Araneae toxins evolution in the context of ecology, and likely facilitate exploration of the popular spider resources in pantropical regions. In the study, the complex toxin repertoire of *H. venatoria* venom is revealed by combining mass spectrum and Sanger sequencing for the venom gland cDNA library. Furthermore, the dynamic evolution of CRPs is explored by combining the data from well-described venom gland ESTs retrieved from ArachnoServer^18,19^. The present results contribute to understanding the evolutionary trends and diversity of CRPs in spider venom.

## Results

### Transcriptomics uncovers the diversity of CRPs in *H. venatoria* venom

From the cDNA library of *H. venatoria* venom gland, 912 sequenced ESTs, and 154 predicted novel CRP precursors were obtained. The CRPs from *H. venatoria* were named according to the nomenclature proposed previously, which describes the toxin’s activity, biological source, and relationship to other toxins^20^. The cDNA sequences of CRPs have been submitted into the public database (http://www.ncbi.nlm.nih.gov/entrez, GenBank accession numbers: KC145575-KC145728). The presence and location of signal peptide cleavage sites in the amino acid sequences were predicted with SignalP 4.1 and SpiderP program (http://www.arachnoserver.org/spiderP.html). A full-length CRPs precursor absolutely contains both a signal peptide and a mature peptide, while some CRPs precursors contain an additional propeptide preceding the mature toxin sequence just as the precursors of most of spider peptide toxins reported before^21^. The alignment of the resultant amino acid sequences revealed extensive variation in the molecular structure of the transcripts for most CRP types.

### Family/cluster identification

In the present study, CRP toxins were classified into 24 families based on the alignment of precursors and their known or predicted cysteine framework. The formation of disulfide bonds stabilizes the three-dimensional (3D) structures of toxins, and is commonly used to classify toxins.

*Family 1–8.* The full primary sequences of the CRPs in the Family 1–8 are comprised of a signal sequence (19–25 residues) and a propeptide (11–19 residues) preceding the mature toxin sequence. The N- and C-terminus of mature peptides are highly variable regions. The 11 members of Family 1 are homologs of Kappa-SPRTX-Hv1c, including its five different precursors (κ-SPRTX-Hv1c_1-5). Signal peptide mode of CRPs in the family is ‘MKh_12_Sh_5_’, where ‘h’ indicates hydrophobic residue, the Arabic numerals denote the number of residues, and capital letters indicate the corresponding amino acids. The propeptides of Family 1 are 19 residues with highly conserved DEQR as an endoproteolytic site preceding the mature peptides, named the Processing Quadruplet Motif (PQM)^22^. Mature peptide mode of Family 1 is ‘XC_I_X_6_C_II_X_5_C_III_C_IV_X_4_C_V_X_3_C_VI_X_4–6_’, where ‘X’ is any amino acid. On the C-terminal of the mature peptides, there is ‘GK’ as the amidation site. The characters of the signal peptides, propeptides and mature peptides of Family 2–8 are compared with those of Family 1, shown in Table 1. According to homology analysis, the mature peptides of Family 1–8 are speculated as the ‘classical’ Inhibitory Cystine Knot (ICK) motif containing three disulfide bonds with I–IV, II–V and III–VI connectivity. The first two disulfide bonds (I–IV and II–V) form an embedded ring which is threaded by the third disulfide bond (III–VI). The backbone regions between successive Cys residues are referred to as loops, numbered starting with loop 1 between Cys I and Cys II^23,24^. There is less amino acid sequence divergence in loop 1 and 3 than in the much more variable loop 2, 4, N- and C-terminus in mature peptides. The precursors of Family 1–7 have higher similarity with those from the same species than others. Only the sequences of CRPs in the Family 8 show high homology with U23-ctenitoxin-Pn1a and U4-agatoxin-Ao1a from *Phoneutria nigriventer* and *Agelena orientalis* respectively (Supplementary Information File 1, Suppl-Fig. 1). Recently, there are also 8 superfamilies of 6-cys ICK motif toxins identified in *Hadronyche infensa*^25^*.*

**Table 1:** Sequence diversity of the predicted 6-cys ICK motif toxins in *H. venatoria.*

Family	Signal peptide mode	Length of propeptides/PQM	Mature peptide mode	C-terminal propeptides
1	MKh_12_S h_5_	19/DEQR	XC_I_X_6_C_II_X_5_C_III_C_IV_X_4_C_V_X_3_C_VI_X_4-6_	G(K)
2	MKT h_11_S h_5_	15/AVAR	X_2_C_I_X_6_C_II_X_5_C_III_C_IV_X_4_C_V_X_3_C_VI_X_3_	G
3	MKh_18_	15/VAAR	X_5_C_I_X_6_C_II_X_6_C_III_C_IV_X_3_C_V_X_3_C_VI_X_5_	GK(K)
4	MKIT h_15_	11/VQAR	XC_I_X_6_C_II_X_6_C_III_C_IV_X_4_C_V_X_4_C_VI_X_6_	GK
5	MKTTh_3_Th_6_Sh_5_	15–17/ATGR	X_3_C_I_X_6_C_II_X_5_C_III_C_IV_X_4_C_V_X_5_C_VI_X	W/O
6	MKTTh_3_Th_6_Sh_5_	15/VTGR	X_2-4_C_I_X_6_C_II_X_5_C_III_C_IV_X_4_C_V_X_5_C_VI_X_3_	RKX_4-5_
7	MKh_5_Th_6_Sh_5_	15/ITVR	X_2-3_C_I_X_6_C_II_X_5_C_III_C_IV_X_4_C_V_X_9_C_VI_X_4_	G
8	MKSSh_7_Th_4_Sh_2_EFTRS	12/VQER	X_2_C_I_X_6_C_II_X_4_C_III_C_IV_X_4_C_V_X_8_C_VI_X_3_	W/O

In the signal peptide mode, ‘h’ indicates hydrophobic residue, and the Arabic numerals denote the number of residues. Capital letters indicate the corresponding amino acids. ‘C’ is cystine, ‘X’ is any residue but cystine, and ‘W/O’ denotes no putative C-terminal propeptides.

**Figure 2:**
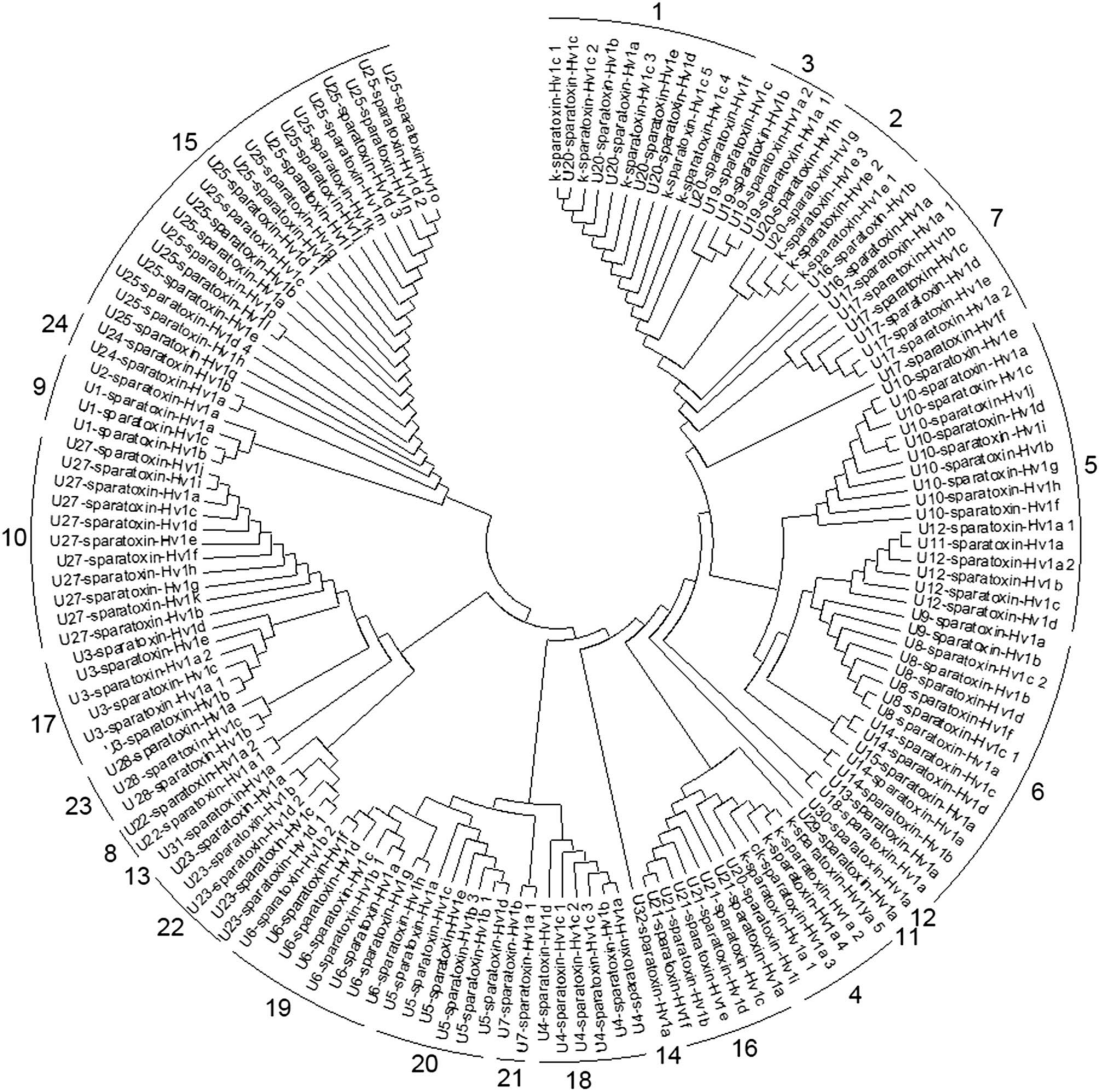
Evolutionary relationships of CRPs from *H. venatoria* venom glands. The evolutionary history was inferred using the Neighbor-Joining method^33^. The tree is drawn to scale, with branch lengths in the same units as those of the evolutionary distances used to infer the phylogenetic tree. The evolutionary distances were computed using the Poisson correction method^34^ and are in the units of the number of amino acid substitutions per site. This analysis involved 151 amino acid sequences. All ambiguous positions were removed for each sequence pair (pairwise deletion option). There were a total of 205 positions in the final dataset. Evolutionary analyses were conducted in MEGA X^35^.

*Family 9.* The precursors of Family 9 have a high content of acidic amino acid in putative mature peptides with a novel Cys scaffold ‘C_I_X_5_C_II_X_3_C_III_X_5_C_IV_XC_V_XC_VI_’. Since no significant homologous sequence has been found in public protein databases, posttranscriptional processes such as alternative splicing or post-translational modifications remain uncertain. In this case, the dotted border indicates the putative short propeptides (Supplementary Information File 1, Suppl-Fig. 2). The motif of Family 9 is the first time reported from spider venom.

**Figure 3:**
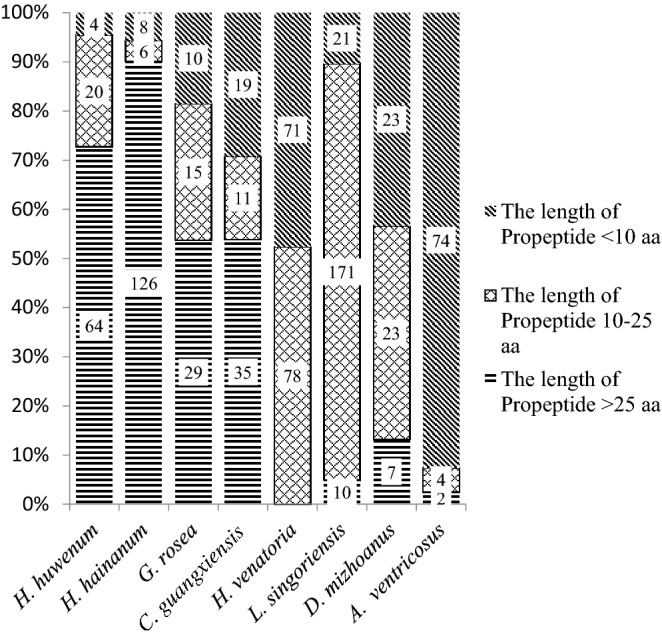
The distribution of the length of propeptide in eight spider species. The species of Mygalomorphae are *C. schmidti, C. hainanus, G. rosea* and *C. guangxiensis*, and the species of Araneomorphae are *L. singoriensis, D. mizhoanus*, *A. ventricosus* and *H. venatoria.* The figure was conducted in Microsoft Excel 2010.

*Family 10.* Family 10 includes eleven homologous sequences which are characterized by two consecutive Cys residues in the middle of signal peptide and that is straight followed by the mature region with a cys-scaffold ‘C_I_X_13_C_II_X_2_C_III_X_12_C_IV_X_3_C_V_X_8_C_VI_’. The scaffold in mature peptide has been identified as a conserved domain pfam01147 (representative proteins gi: 221468699, 5921747 shown in Supplementary Information File 1, Suppl-Fig. 3), which includes all known crustacean hyperglycemic hormones (CHHs) found in the sinus gland of isopods and decapods^26^ and the molt-inhibiting hormone (MIH) of the lobster *Homarus americanus*^27^. The three disulfide bridges are C_I_–C_V_, C_II_–C_IV_, and C_III_–C_VI_^28^. In addition, the amino acid sequences of several translated cDNA (gi: 304306070, 304307035, 304306844, 304306583) from *Loxosceles intermedia* venom gland library^29^ are also similar to that of Family 10 (Supplementary Information File 1, Suppl-Fig. 3). The latrodectins which are identified in widow spider venom glands, also share six conserved cysteines that adopt the same disulfide bond pairing in the mature peptide^30,31^.

**Figure 4:**
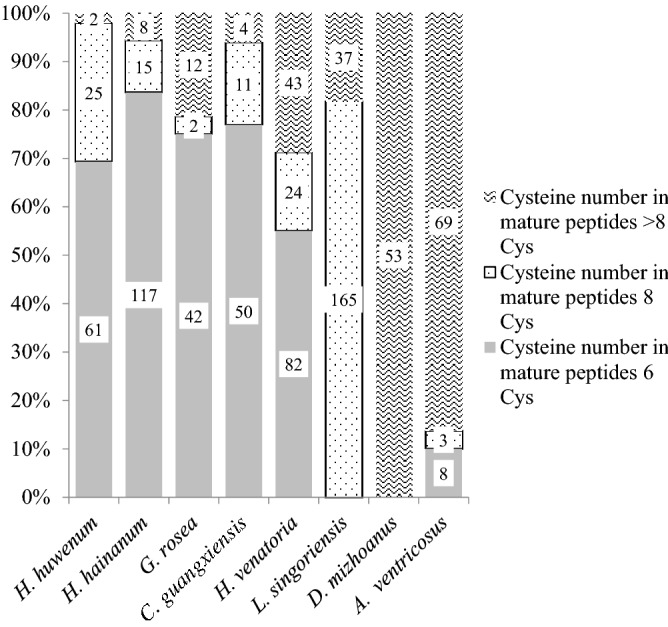
The distribution of cysteines in mature peptide in eight spider species. The species of Mygalomorphae are *C. schmidti, C. hainanus, G. rosea* and *C. guangxiensis*, and the species of Araneomorphae are *L. singoriensis, D. mizhoanus*, *A. ventricosus* and *H. venatoria*. The figure was conducted in Microsoft Excel 2010.

*Family 11, 12, 13 and 14.* The four families have eight cysteines with a typical motif ‘C_I_X_6_C_II_X_n_C_III_C_IV_X_4_C_V_XC_VI_X_n_C_VII_XC_VIII_’ where X is any residue but cystine. However, the amounts and properties of residues in the loops between C_II_ and C_III_, C_VI_ and C_VII_ and at the C-terminus vary greatly. The sequences of the signal and precursor proteins, as well as endoproteolytic sites, are also diverse. In Family 14, there is a long loop between C_VI_ and C_VII_ and a very short propeptide preceding the mature region. Significantly, there is no propeptide predicted in the precursor of U32-sparatoxin-Hv1a. The amino acid sequences of Family 11, 12, 13, and 14 are aligned with the most similar known homologs (Supplementary Information File 1, Suppl-Fig. 4). The cysteine-frame is also employed by superfamily 3 and 23 of *H. infensa* venom. The eight cysteines are arranged in four disulfide bonds (C_I_–C_IV_, C_II_–C_V_, C_III_–C_VIII_, C_VI_–C_VII_), which form an extended ICK motif^25^.

*Family 15.* Family 15 includes 20 unique sequences that are highly homologous. It is noteworthy that Family 15 includestranscripts coding a new venom peptide type with a high mRNA expression level in *H. venatoria.* There are 20 orthologs identified coding full-length cysteine frame in Family 15. The transcripts of U25-sparatoxin-Hv1c and U25-sparatoxin-Hv1j are 30 and 29 copies, respectively, which are the top two precursors identified in the *H. venatoria* venom cDNA library. There are twelve residues between the signal peptides and the first Cys, but no usual PQM. The mature region is characterized by a novel eight-Cys scaffold ‘C_I_X_21_C_II_X_4_C_III_X_9_C_IV_X_10_C_V_X_11_C_VI_C_VII_X_4_C_VIII_’. The transcript of a secretory protein with the identical Cys-frame has been identified from the black widow spider (gi: 318087504). However, it is hypothesized to be involved in wrapping silk fibers. Moreover, several hypothetical non-secretory proteins from *Amblyomma maculatum* also adopt the same eight-Cys framework (Supplementary Information File 1, Suppl-Fig. 5).

*Family 16.* The six precursors are highly homologous with Omega-agatoxin-1A (gi: 2507406) from *Agelenopsis aperta* containing a ten-Cys scaffold ‘C_I_X_6/8_C_II_XC_III_X_6_C_IV_XC_V_X_7/11_C_VI_XC_VII_X_7_C_VIII_X_5_C_IX_X_19/20_C_X_’, so they belong to the omega-agatoxin superfamily, which has a particularly exciting feature of the prepropeptide with the occurrence of two glutamate-rich sequences interposed between the signal sequences, the major peptide toxin, and the minor toxin peptide. The heterodimer of the two subunits is linked by a disulfide bond^32^ (Supplementary Information File 1, Suppl-Fig. 6).

*Family 17 and 18.* Family 17 is homologous with U19-ctenitoxin-Pn1a (gi: 50401390), Hainantoxin-XIV-7 (gi: 310946827), HWTX-XIVa2 (gi: 166007861) precursor, and a toxin-like peptide (gi: 380692240) from *G. rosea*. Family 18 is homologous with U3-aranetoxin-Ce1a (gi: 27805756). The precursors in both families contain a signal peptide and a mature peptide with a ten-Cys-scaffold -like ‘C_I_X_n_C_II_X_4_C_III_C_IV_X_n_C_V_X_9_C_VI_X_n_C_VII_XC_VIII_X_5_C_IX_X_n_C_X_’. However, the residues are very different in the loops between cysteines. The loops of C_IV_–C_V,_ C_VI_–C_VII,_ and C_IX_–C_X_ are longer in Family 18 than those in Family 17 (Supplementary Information File 1, Suppl-Fig. 7). Recently, the ten-Cys scaffold was reported as superfamily 2 in Australian funnel-web spiders *H. infensa*, named mamba intestinal toxin 1 (MIT1)-like toxin. The precursors of SF2 also have been identified with no propeptide region^25^.

*Family 19, 20 and 21.* All the three families are composed of a signal peptide and a mature region with a ten-Cys framework ‘C_I_X_7_C_II_X_8_C_III_C_IV_X_4_C_V_X_5_C_VI_C_VII_X_3_C_VIII_X_3_C_IX_X_17_C_X_’, which has a high degree of similarity to the Cysteine frame of U7-agatoxin-Ao1a (gi: 74845728) and U20-lycotoxin-Ls1a/c/d (gi: 313471673/313471696/313471677) from *A. orientalis* and *L. singoriensis* respectively. The amino acids in the loop between C_VIII_ and C_IX_ are conserved in the peptides even from different spider families (Sparassidae, Agelenidae and Lycosoidea). The sequences between C_IV_ and C_V_ are also conserved in the three families from the same spider *H. venatoria.* However, those in other spaces are less homologous, especially, the aminoacid sequences in the N- and C-terminus are much various and diverse in Family 19, 20, and 21 (Supplementary Information File 1, Suppl-Fig. 8).

*Family 22.* The predicted peptide sequences in Family 22 have a similar disulfide bonding pattern and structure to U9-agatoxin-Ao1a (gi: 74845712). Their typical Cys bonding pattern is ‘C_I_X_6_C_II_X_3_C_III_XC_IV_C_V_X_5_C_VI_XC_VII_X_4_C_VIII_XC_IX_X_8_C_X_X_6_C_XI_X_12_C_XII_’. However, the sequences are much different in signal peptides, propeptides, and loops between the cysteines, and PQM is apparent in U9-agatoxin-Ao1a, rather than in the precursors of Family 22 from *H. venatoria* (Supplementary Information File 1, Suppl-Fig. 9).

*Family 23.* There are three secretory proteins with a long 12-Cys framework in Family 23 as follows: ‘C_I_X_7_C_II_X_23_C_III_X_9_C_IV_X_7_C_V_X_22_C_VI_X_15_C_VII_X_11_C_VIII_X_11_C_IX_X_8_C_X_X_8_C_XI_X_22_C_XI_’. The precursors had no homologs when they were aligned against the Database of GenBank, EMBL, and DDBJ. However, two sequences from the spider EST database were matched using TBLASTN, which have not been identified as toxins. The amino acid sequences of gi: 304306221 and gi: 189216028, which are in the cDNA library from *L. intermedia* venom gland and *Acanthoscurria gomesiana*, respectively, are homologous to U28-sparatoxin-Hv1a with 43% (E-value is 5e−07) and 48% (E-value is 1e−06) positives (Supplementary Information File 1, Suppl-Fig. 10).

*Family 24*. The two precursors in Family 24 with only two cysteines ‘C_I_X_6_C_II_X_16_′ in the mature region have no significant sequence homolog in the Database of GenBank, EMBL, and DDBJ. The propeptides were predicted by using SpiderP (Supplementary Information File 1, Suppl-Fig. 11). Noteworthy, there are two different probable cleavage modes.

### Mass spectrometry reveals complex PTM in spider peptide toxins

The peptide elution from RP-HPLC separation was collected and analyzed by MALDI-TOF mass spectrometry. There are several distinct components in each eluent on the retention time (RT). About 140 different peptide masses were detected, most of which fall in the 2800–5000 Da mass range and only 19 between 5000 and 7000 Da. Given the low abundance peptides may vanish in the process of isolation and purification, the crude venom was directly analyzed by MALDI-TOF mass spectrometry and resulted in 227 peptide masses, a considerable part of which are in the 5000–8000 Da mass range. Intriguingly, only a few of peptide masses (22) match to the theoretical molecular weights directly even though the disulfide bonds and C-terminal amidation are considered (Supplementary Information File 2 and Supplementary Information File 3). Significantly, there are many abundant long precursors cannot match to any mass, which suggests that the post-translational modifications are prevalent and to be unscrambled in *H. venatoria* venom. There are several characters observed: Firstly, the C-terminal loops of most CRPs precursors (101 out of 154) are ≥ 5 amino acid residues. Secondly, the equivocal hydrolysis sites of propeptide are not the usual motifs recognized by SpiderP. For example, U1-sparatoxin-Hv1c and U23-sparatoxin-Hv are predicted with a long N-terminal loop (> 22 amino acids), respectively. Both the long C-terminal and N-terminal loops have more probabilities for processing. Thirdly, the propeptide occurs between the cysteines. For example, the precursor of U21-sparatoxin-Hv1a is speculated under post-translational proteolytic processing by proprotein convertases at two sites, one (EQAR) following the signal peptide, and the other (REEDELER) between the ninth and tenth cysteines (Supplementary Information File 1, Suppl-Fig. 6).

### Phylogenetic study of the CRPs in* H. venatoria*

The 151 precursor sequences of CRPs from *H. venatoria* venom gland were aligned using Clustal X 2.0. The resulting alignment was imported into MEGA X software to construct the phylogenetic tree with the neighbor-joining method. All ambiguous positions were removed for each sequence pair (pairwise deletion option). There were a total of 205 positions in the final dataset. Most of the 6-cys ICK motif precursors (Family 1, 2, 3, 5, 6, and 7) were defined as the relatively original clade. Only Family 4 and 8, with shorter propeptides (12 aa), were placed outside the “older” clade. Remarkably, Family 8, whose signal peptides are different from and longer than other families with speculative 6-cys ICK motif, was located far away from the original clade precursors (Family 1, 2, 3, 5, 6, and 7). Intriguingly, four 8-cys ICK-like motif families (Family 12, 13, 14 and 15) were put in different clades. It is reasonable to put them in four families, although the cys-scaffold of the mature peptide looks similar. Family 17 and 18, which both adopt a ten-Cys-frame ‘C_I_X_n_C_II_X_4_C_III_C_IV_X_n_C_V_X_9_C_VI_X_n_C_VII_XC_VIII_X_5_C_IX_X_n_C_X_’ in the mature peptide domains, were also arranged in two far-away phylogenetic clades (Fig. 2). The three loops (C_IV_–C_V_, C_VI_–C_VII,_ and C_IX_–C_X_) are longer in Family 18 than in Family 17.

The evolutionary selection of each Family was also conducted in MEGA X. The sequences of each family of *H. venatoria* venoms were analyzed and aligned, and the gap positions were omitted in the subsequent analysis. The nucleotide sequences of signal peptides, propeptides, and mature peptides of Family 1–10 and 15–23 were compared, respectively. The original Nei-Gojobori model (p-distance) was used to estimate the number of synonymous substitutions (Ds) of each synonymous site, the number of non-synonymous substitutions (Dn) of each non-synonymous site, and calculate ω (Dn/Ds) values. The *p*-value of each family was calculated and analyzed (Table 2).

**Table 2:** Evolution analysis of each family of *H. venatoria* venoms.

Gene name	ω			*p*-value		
	Signal	Pro	Mature	Signal	Pro	Mature
Family 1	0.478615148	0.510231237	0.332445114	0.005929225	**0.940316982**	0.000128405
Family 2	0	0	0	1.65377E−11	7.03411E−08	2.9917E−12
Family 3	0.049919485	0	5.008187135	0.095276259	7.03411E−08	**0.110095282**
Family 4	0.095238095	0	0.125	**0.433820531**	7.03411E−08	0.000117168
Family 5	0.195828255	0	0.241523218	**0.625979649**	7.03411E−08	5.29103E−05
Family 6	0.090301003	1.489437121	1.125851979	0.000295395	0.006537796	3.61297E−06
Family 7	0.452407683	1.188707564	0.922396864	0.027520806	**0.125681993**	0.044007869
Family 9	0.527353422	0.788144231	0.422801919	0.000274795	0.046929982	0.004300409
Family 10	0	–	0.425	1.65377E−11	–	**0.23625743**
Family 15	0.014619883	–	0.840435754	3.35828E−10	–	**0.184069688**
Family 16	0	0.497297297	0.22173913	1.65377E−11	**0.873999133**	0.000927842
Family 17	0	–	0.113043478	1.65377E−11	–	0.010813093
Family 18	0.3	–	0.255672269	**0.721660871**	–	0.005900781
Family 19	0.487702917	–	0.44471645	0.010113167	–	0.046432457
Family 20	0.427655367	–	2.154982007	0.067588108	–	**0.109231216**
Family 21	0	–	0	1.65377E−11	–	2.9917E−12
Family 22	0	0.240072202	0.26	1.65377E−11	**0.333780885**	0.054726703
Family 23	0	0	0	1.65377E−11	7.03411E−08	2.9917E−12

The bold background cell represents *p*-value ≥ 0.1.

The ω values of both pro and mature peptide coding genes are over 1.0 only in Family 6, which suggests that the rate of non-synonymous mutation is significantly higher than that of synonymous mutation, and positive selection may increase the diversity of venoms in Family 6. The ω values of signal peptide, propeptide and mature peptide coding genes are under 1.0 with *p*-value ≤ 0.05 in almost all CRPs families except Family 6, which means most of CRPs are under negative selection. We also noticed that the ω values of some families are unreliable as their *p*-value ≥ 0.05, which may attribute to very few samples.

### Common types of spider venom CRPs from multiple families

In the present study, the spider CRPs discovered with Sanger sequencing and Edman degradation sequencing methods were gathered from Arachnoserver spider toxins database^18^. There are more than 28 scaffolds of the CRPs from 19 families, 55 species of spiders. The short CRPs with 6-cys are popular in Mygalomorphae, which present account for 82.5% and mainly include three kinds of scaffolds (ICK, disulfide-directed β-hairpin (DDH) and Kunitz). Relatively, the 6-cys peptides account for 22.2% in Araneomorphae which are mainly ICK-like motif peptides, a few CHH and low molecular weight protein (LMWP) motifs. The long CRPs with more than 6 cysteines account for 17.5% in Mygalomorphae, which present six scaffolds with 8-cys and one with 10-cys. However, the long CRPs are much more popular in Araneomorphae, which present account for 77.8% and include diversified scaffolds (Table 3).

**Table 3:** Characteristics and distribution of common motifs of CRPs in Mygalomorphae and Araneomorphae.

	Mygalomorphae	Araneomorphae
Total number of 6-Cys CRPs/percentage	597/82.5%	140/22.2%
Total number of > 6-Cys CRPs/percentage	127/17.5%	520/77.8%
Main motifs = 6-Cys/ number	ICK-like/476 DDH/99 Kunitz/22	ICK-like/118 CHH/20 LMWP/2
Main motifs > 6-Cys/ number	C…C…CXCC…C…C…C/36 C…C…CCC…C…C…C/6 C…C…C…CC…C…C…C/2 C…C…CC…CC…C…C/9 C…C…CC…C…C…C…C/15 C…C…CC…CXC…CXC/23 C…C…CC…C…C…CXC…C…C/35 CRISP > 14C/1	C…CXC…CXC…CXC…C/5 C…CXC…CXC…CXC…C…C…C/12 C…CXC…CXC…C…C…C/1 C…C…CC…C…CC…C…C…C/74 C…C…CC…C…C…CXC/1 C…C…CC…CXC…CXC/254 C…C…CC…C…C…CXC…C…C/38 C…CXC…CC…CXC…CXC…C/1 C…C…CXCC…CXC…CXC…C/34 C…C…CXCC…CXC…CXC…C…C…C/18 C…C…CXCC…C…C…CXC…C…C…C/7 C…C…CXCC…C…C…C…C…C/12 C…C…C…C…C…C…CXC…C…C/2 C…C…C…CXC…CXC…C…C…CXC…C/24 C…C…CXCCXC…CXC…CXC…C…C/8 C…C…CC…CXC…C…C…CXC…CXC/7 C…C…CXCC…CXC…CXC…C…C…C…C…C/7 C…C…C…C…C…C…C…C…C…C…C…C/3 CRISP > 14C/14

Capital letters indicate the corresponding amino acids. ‘C’ is cystine, and ‘X’ is any residue but cystine. The sequences are identified by deducing from Sanger sequencing and/or by Edman sequencing.

### Evolution trend analyses of the propeptides of CRPs and cysteines in mature peptide

The high-quality cDNA libraries and full-length EST sequences from eight spiders, 4 of Mygalomorphae and 4 of Araneomorphae, were used for the analysis of propeptide and cysteines in the mature domain. The length of propeptide varies among the species. There are longer propeptides in Mygalomorphae than in Araneomorphae. The propeptides longer than 25 aa account for 72.7%, 90%, 53.6% and 53.8% in *C. schmidti*, *C. hainanus, G. rosea* and *C. guangxiensis*; respectively. By contrast, there are only 5%, 13.2% and 2.5% propeptides longer than 25 aa in *L. singoriensis, D.mizhoanus* and *A.ventricosus,* respectively, and none is longer than 25 aa in *H. venatoria*. By contrast, the percentages of precursors with a propeptide less than 10 residues are 4.6%, 5.7%, 17.8% and 29.2% in *C. schmidti, C. hainanus, G. rosea* and *C. guangxiensis,* respectively, and 47.7%, 43.4% and 92.5% in three species (*H. venatoria, D. mizhoanus* and *A. ventricosus*) of Araneomorphae. Although the ratio percentage of precursors with the shortest propeptide is only 10.4% in *L. singoriensis*, the precursors with a 10–25 aa propeptide account for 84.7% (Fig. 3).

As for the cysteines in the mature domain, there are 69.3%, 83.6%, 75%, 76.9% peptides with 6-cys motif in the four species of Mygalomorphae (*C. schmidti, C. hainanus, G. rosea* and *C. guangxiensis*), respectively. However, there is no 6-cys CRPs described in *L. singoriensis* and *D. mizhoanus* so far and 55% and 10% peptides with 6-cys motif in *H. venatoria* and *A. ventricosus*, respectively. On the contrary, there are more peptides with ≥ 8-cys motif, and the ratios percentages are 45%, 100%, 100% and 90% in *H. venatoria, L. singoriensis, D. mizhoanus* and *A. ventricosus,* respectively (Fig. 4).

The styles of cysteine frames of CRPs with more than 6 cysteines in Mygalomorphae are much fewer than those in Araneomorphae. The evolution of CRPs expressed in the venom of *H. infensa*, a spider in Mygalomorphae, was recently shown largely by duplication and elaboration of a single ancestral knottin gene^25^. However, the CRPs in the venom of *H. venatoria,* a spider in Araneomorphae, evolved by duplication as well as recruitment. In the present study, Family 1–8, 11–14, 17 and 18 are predicted to comprise of simple or highly derived knottins and evolve by multiple duplications of an ancestral ICK gene followed by periods of diversification, which is the similar style employed by CRPs in funnel-web spider venom^25^. Furthermore, ten families with different cysteine frame predicted beyond the ICK scaffold are also identified in the venom of *H. venatoria.* Among them, four novel cysteine scaffolds (Family 9, 15, 23 and 24) are uncovered for the first time in spider venom. The homogeneous analyses indicate that CRPs in both Family 15 and Family 10 of *H. venatoria* are evolved by recruitment events from non-toxin genes followed by limited extensive duplication. Our study overall contributes to the understanding of the molecular diversity and combinatorial evolutionary trends of CRPs in spider toxins.

## Discussion

The construction of the cDNA library with ESTs Sanger sequencing approach has been proved to be a rapid and reliable method for discovering new genes and obtaining data on the gene expression of CRPs in venom glands, which are characterized as multigene displaying high similarity in part of their sequence^36^. In our group, second-generation sequencing technologies were applied to explore the diverse peptide toxins in venom of *C. schmidti*^37^ and *Latrodectus tredecimguttatus*^38^, the sequencing assembly of which strongly relied on the ESTs Sanger sequencing data. Furthermore, the data from the Sanger sequencing method usually include the information of 5′ and 3′ untranslated regions which are very important for evolutionary analysis^39^. Also, the EST clones facilitate subsequent function research, for example, recombinant expression, transgene, gene modification, etc. Since the transcript of *H. venatoria* venom gland was investigated for the first time, the traditional Sanger sequencing technology was employed. In the present study, 154 transcripts coding CRP precursors in 24 families are uncovered, among which, there are 8 families of short ICK toxins with 6-cys, 2 novel 6-cys non-ICK motifs, and 13 families of long CRP peptides with 8, 10 and 12-cys. Intriguingly, four novel cysteine scaffolds transcripts (Family 9, 15, 23 and 24) are first described in spider venom. A few of ICK-like peptides can match to a molecular mass with considerations of normal PTM such as the disulfide bonds and C-terminal amidation (Supplementary Information File 2). The diversity of primary structure within the *H. venatoria* venom families suggests that there have been few evolutionary restraints on CRPs diversification outside of the disulfide bridges that direct the 3D fold of these peptides. These findings highlight the extensive diversity of CRPs in *H. venatoria* venoms which can not only provide important data for evolutionary analysis of CRPs in spider venom but also be exploited as novel therapeutic and biopesticide lead molecules.

In the present study, CHHs-like peptide genes are identified in Family 10 from the venom gland of *H. venatoria*, and that also widely distributed in very distantly related families: *Agelenidae*, *Sicariidae*, *Theridiidae*, *Sparassidae*, *Pisauridae* and *Nephilidae*. It is strongly suggested that CHHs-like peptides are derived from the superfamily of neuropeptides containing Crustacean Hyperglycemic Hormones (CHH) and independently recruited in spider venom glands^31^. The structure of U1-agatoxin-Ta1a from *Eratigena agrestis* was determined using heteronuclear NMR as a structural homolog of the CHH family recently, which confirms the molecular evolutionary analyses indicating that CHHs-like toxins are highly derived members of the ITP/CHH family^40^. The hormone-derived venom peptides, named HAND toxins, are among the most stable peptides ever described, which provide a novel molecular scaffold for engineering drugs and insecticides^40^. Family 15 from the venom gland of *H. venatoria* with a novel cysteine scaffold in the mature region is also seemly evolved by recruitment of genes encoding normal body proteins followed by extensive duplication and neofunctionalization to play a role in killing and paralyzing prey or defending the organism. A secretory protein with the identical Cys-bone structure has been identified from black widow spider (gi: 318087504). However, it is noted as a gene involved in coding silk fibers. Moreover, several hypothetical non-secretory body proteins from *A. maculatum* also adopt the same eight-Cys-framework (Supplementary Information File 1, Suppl-Fig. 5) without a predicted signal peptide. Therefore, two clusters of transcripts were detected in *H. venatoria* venom gland EST library which showed similarity to previously reported non-venom proteins.

Spiders evolved over some 300 million years, and have become the most diverse terrestrial organism group only after insects. Spiders have evolved efficient weapons represented by the venom and/ or the silk for their hunting strategies. The venom system has evolved to restrain prey, defend and deter competitors. However, spiders investigated for venom molecular research are not more than 0.5% of all known species so far^11^, and have focused on many big species and medically important species. With the evolution of the spider from Mygalomorphae to Araneomorphae the spider body size is evolving smaller and the predators have evolved to adopt webs as capture strategies. The species hunting without silk is considered more offensive, and its venoms often show higher complexity and potency^41^. A recent study suggested that peptides (2–15 kDa) in spider venoms are mainly responsible for the paralysis efficacy of the venom^42^. In the present study, *H. venatoria* in the family Sparassidae, a relatively primitive species of Araneomorphae, does not directly use web or silk to capture prey that is similar in a way to the predation tactics of cursorial spiders *Lycosidae*, *Hexathelidae* and *Theraphosidae*. It is an important candidate for the evolutionary investigation of CRPs in spider venom. The transcript data of *H. venatoria* venom gland along with before work about spider peptide toxins in our group and high-quality cDNA library data in the public database provide the opportunity to take a holistic view analysis of the evolution of spider venom CRPs. According to the analysis of the sequences gathered from Arachnoserver spider toxins database, the short CRPs with 6-cys account for at least 69% of all discovered CRPs in each of the four species of Mygalomorphae (*C. schmidti, C. hainanus, G. rosea* and *C. guangxiensis*) (Fig. 4), and mainly include three kinds of scaffolds (ICK, DDH and Kunitz)^18^. By contrast, in Araneomorphae, there is no 6-cys CRP in *L. singoriensis* and *D. mizhoanus*. Both belong to the superfamily Lycosoidea (Lycosidae and Dolomedes respectively), which are most evolutionary at the distant end in the spider system. The 6-cys CRPs are 10% and 55% in *A. ventricosus* and *H. venatoria* respectively. *A. ventricosus* mainly uses webs for predation, which may explain the low percentage of 6-cys CRPs in this species. Intriguingly, the 6-cys CRPs in *H. venatoria* have a much shorter propeptide comparing to that in Mygalomorphae. The propeptide that was ever thought of as a necessary part of a spider CRPs precursor is short, even disappears in several families of CRPs in Araneomorphae venom.

Overall, the present study shows the high diversity of CRPs in *H. venatoria* and suggests the evolutionary trends of CRPs in spider venom from Mygalomorphae to Araneomorphae: the mature peptides have been developed longer with more cysteines; the propeptides have diminished and even vanished; and the CRPs evolved by multiple duplications as well as recruitments of non-toxin genes.

## Methods

### Spider collection

The spiders of *H. venatoria* were collected by sweeping and visually searching from the old buildings in the farm without pesticides for scientific research of Hunan Agricultural University, Changsha, China (28° 18′ 33′′ N, 113° 07′ 69′′ E).

### Separation of venom peptides by HPLC

The venom was collected using the method of electrical milking. Crude venom was suspended in deionized water, centrifuged (15,000 *g*, 4 °C, 10 min), filtered on 0.45 mm microfilters, lyophilized, and stored at − 20 °C for further analysis. 5 mg of lyophilized venom was loaded onto an analytical Vydac C18 RP HPLC column (218TP54, 4.6 × 250 mm^2^) and eluted at the flow rate of 1 mL/min using a gradient of 0–20% buffer B (0.1% v/v trifluoroacetic acid [TFA] in acetonitrile [ACN]) over 5 min followed by a gradient of 20–40% buffer B over 50 min and 45–60% buffer B over 10 min (Buffer A was 0.1% v/v TFA in water). The peptide elution was monitored at 215 nm and 280 nm. The fractions were manually collected.

### Venom peptide identification by mass spectrometry

The peaks eluted from RP HPLC were collected for MALDI-TOF mass spectrometry analysis (UltraFlex I, Bruker Daltonics). The eluted fractions (1 μL) were loaded on a 384-well target plate along with an equal volume of a matrix solution containing 20 mg/mL R-cyano-4-hydroxycinnamic acid (CHCA), 50% ACN, and 0.1% TFA. The mixture was allowed to dry at room temperature. Calibration of the instrument was performed externally with a peptide calibration standard II (Bruker, Germany). Mass spectrometry was performed using an acceleration voltage of 25 kV. After desalting with ZipTips (C4), 1 μL of cleaned crude venom was subjected to MALDI-TOF MS analysis.

### cDNA library construction and expression sequence tag sequencing

The cDNA library was prepared and sequenced as previously described^43^. Simply, eight adult female spiders were aggravated to secret their venom gland contents and encourage the production of venom transcripts^44^. Four days later, the venom glands of the eight spiders were harvested and homogenized in liquid nitrogen with the presence of TRIzol reagent (Invitrogen). Polyadenylic acid (+) [polyA(+)]-containing RNAs were purified from the total RNA on oligo(dT)-cellulose affinity column using the mRNA Purification Kit (Promega) according to the manufacturer’s protocol. The full-length cDNA library was constructed using Primer Extension protocol as described in the Creator SMART cDNA Library Construction Kit (Clontech). The polymerase chain reaction was performed with the M13 forward and reverse primers from the kit to rapidly screen recombinant clones. The clones containing inserts ≥ 500 base pairs were grown in LB medium containing chloramphenicol (30 mg/mL) in 96-well plates for 16 h. The plasmids were extracted by alkaline lysis and sequenced from the 5′-end on an automated ABI PRISM 3700 sequencer (Perkin Elmer) using the T7 promoter primer and ABI PRISM Big Dye terminator v3.1 ready reaction cycle sequencing kit (Applied Biosystems).

### CRPs identification and evolutionary analyses

The sequenced cDNA was trimmed by removal of vector, primer sequences and poly(A) tails with ABI PRISM DNA Sequencing Analysis Software V.3.3^45^. The consensus sequences of each cluster were further filtered by screening for homology to ribosomal RNA, mitochondrial DNA and *E. coli* genome sequences^46^. After deleting matches, the remaining sequences were searched against public databases (nr/NCBI, SwissProt/UniProtKB and TrEMBL/UniProtKB) using the BLASTn or BLASTx programs to identify putative functions of the new expression sequence tags (ESTs)^47^. The signal peptides were predicted with the SignalP 4.1 program (http://www.cbs.dtu.dk/services/SignalP/) ^48^ and SpiderP (http://www.arachnoserver.org) ^49^. Furthermore, the putative CRPs were searched in the KNOTTIN database (http://knottin.cbs.cnrs.fr) ^50,51^. Multiple sequences of precursors were aligned using the ClustalW program^52^. The resulting alignments were then hand-edited using the BioEdit program (http://www.mbio.ncsu.edu/BioEdit/BioEdit.html). Sequences were aligned using Clustal X 2.0, and gapped positions were omitted from subsequent analyses. The original nei-gojobori model (p-distance) of MEGA7.0 software was used to estimate the number of Ds for each synonymous site and the number of Dn for each non-synonymous site, and ω was calculated^53^. The resulting alignments were imported into MEGA X software to construct a phylogenetic tree with the neighbor-joining method^54,55^. The 64-bit Microsoft @excel @2019 MSO (16.0.12730.20342) was used to calculate and analyze the p-value of each family.

## Supplementary Information

Supplementary Information 1.

Supplementary Information 2.

Supplementary Information 3.

## Data Availability

The cDNA sequences of CRPs have been submitted into the public database (http://www.ncbi.nlm.nih.gov/entrez, GenBank accession numbers: KC145575-KC145728). This article is partly present on a website and can be accessed on https://www.researchsquare.com/article/rs-24220/v1. This article is not published nor under publication elsewhere.

## References

[1] Fry BG (2009). The toxicogenomic multiverse: convergent recruitment of proteins into animal venoms. Annu. Rev. Genom. Hum. Genet..

[2] Gracy J, Chiche L (2011). Structure and modeling of knottins, a promising molecular scaffold for drug discovery. Curr. Pharm. Des..

[3] Berkut AA (2015). Structure of membrane-active toxin from crab spider *Heriaeus melloteei* suggests parallel evolution of sodium channel gating modifiers in Araneomorphae and Mygalomorphae. J. Biol. Chem..

[4] King GF, Hardy MC (2013). Spider-venom peptides: structure, pharmacology, and potential for control of insect pests. Annu. Rev. Entomol..

[5] Zhu S (2011). Molecular diversity and functional evolution of scorpion potassium channel toxins. Mol Cell Proteom..

[6] Garb JE, Hayashi CY (2013). Molecular evolution of alpha-latrotoxin, the exceptionally potent vertebrate neurotoxin in black widow spider venom. Mol. Biol. Evol..

[7] He Y (2013). Molecular diversity of *Chaerilidae venom* peptides reveals the dynamic evolution of scorpion venom components from Buthidae to non-Buthidae. J. Proteom..

[8] Junqueira-de-Azevedo IL (2015). Venom-related transcripts from *Bothrops jararaca* tissues provide novel molecular insights into the production and evolution of snake venom. Mol. Biol. Evol..

[9] Puillandre N (2014). Molecular phylogeny and evolution of the cone snails (Gastropoda, Conoidea). Mol. Phylogenet. Evol..

[10] Vassilevski AA, Kozlov SA, Grishin EV (2009). Molecular diversity of spider venom. Biochemistry (Mosc).

[11] Kuhn-Nentwig L, Stocklin R, Nentwig W (2011). Venom composition and strategies in spiders: is everything possible?. Adv. Insect Physiol. Spider Physiol. Behav. Physiol..

[12] Sugumaran MP, Kumar MG, Ramasamy K, Vincent S (2004). Observations on life cycle of certain spiders from Western Ghats of Tamil Nadu. J. Environ. Biol..

[13] Edwards GB (2009). Huntsman Spider, *Heteropoda venatoria* (Linnaeus) (Arachnida: Araneae: Sparassidae). Florida Cooperative Extension Service.

[14] Huang Y (2017). Peptide-rich venom from the spider *Heteropoda venatoria* potently inhibits insect voltage-gated sodium channels. Toxicon.

[15] Gray MR (2010). A revision of the Australian funnel-web spiders (Hexathelidae: Atracinae). Rec. Aust. Mus..

[16] Zhu, Y. *Comparison and Analysis of Four Cytotoxic Peptides from the Venom of Spider Lycosa Singoriensis.* Master thesis, Hunan Normal University (2011).

[17] Paquin P, Dupérré N (2003). Guide d'identification des Araignées (Araneae) du Québec. Int. J. Mech. Ences.

[18] Herzig V (2011). ArachnoServer 2.0, an updated online resource for spider toxin sequences and structures. Nucl. Acids Res.

[19] Pineda SS (2018). ArachnoServer 3.0: an online resource for automated discovery, analysis and annotation of spider toxins. Bioinformatics.

[20] King GF, Gentz MC, Escoubas P, Nicholson GM (2008). A rational nomenclature for naming peptide toxins from spiders and other venomous animals. Toxicon.

[21] Escoubas P (2006). Molecular diversification in spider venoms: a web of combinatorial peptide libraries. Mol. Divers..

[22] Kozlov S (2005). A novel strategy for the identification of toxinlike structures in spider venom. Proteins.

[23] Norton RS, Pallaghy PK (1998). The cystine knot structure of ion channel toxins and related polypeptides. Toxicon.

[24] Craik DJ, Daly NL, Waine C (2001). The cystine knot motif in toxins and implications for drug design. Toxicon.

[25] Pineda SS (2020). Structural venomics reveals evolution of a complex venom by duplication and diversification of an ancient peptide-encoding gene. Proc. Natl. Acad. Sci. USA.

[26] Davey ML (2000). Five crustacean hyperglycemic family hormones of *Penaeus monodon*: complementary DNA sequence and identification in single sinus glands by electrospray ionization-Fourier transform mass spectrometry. Mar. Biotechnol. (NY).

[27] Aguilar MB, Falchetto R, Shabanowitz J, Hunt DF, Huberman A (1996). Complete primary structure of the molt-inhibiting hormone (MIH) of the Mexican crayfish *Procambarus bouvieri* (Ortmann). Peptides.

[28] Katayama H (2003). The solution structure of molt-inhibiting hormone from the Kuruma prawn *Marsupenaeus japonicus*. J. Biol. Chem..

[29] Gremski LH (2010). A novel expression profile of the *Loxosceles intermedia* spider venomous gland revealed by transcriptome analysis. Mol. Biosyst..

[30] Gasparini S (1994). The low molecular weight protein which co-purifies with alpha-latrotoxin is structurally related to crustacean hyperglycemic hormones. J. Biol. Chem..

[31] McCowan C, Garb JE (2014). Recruitment and diversification of an ecdysozoan family of neuropeptide hormones for black widow spider venom expression. Gene.

[32] Santos AD (1992). Heterodimeric structure of the spider toxin omega-agatoxin IA revealed by precursor analysis and mass spectrometry. J. Biol. Chem..

[33] Saitou N, Nei M (1987). The neighbor-joining method: a new method for reconstructing phylogenetic trees. Mol. Biol. Evol..

[34] Kaur G, Iyer LM, Subramanian S, Aravind L (2018). Evolutionary convergence and divergence in archaeal chromosomal proteins and chromo-like domains from bacteria and eukaryotes. Sci. Rep..

[35] Kumar S, Stecher G, Li M, Knyaz C, Tamura K (2018). MEGA X: molecular evolutionary genetics analysis across computing platforms. Mol. Biol. Evol..

[36] Kaas Q, Craik DJ (2015). Bioinformatics-aided venomics. Toxins (Basel).

[37] Zhang Y (2014). Toxin diversity revealed by a transcriptomic study of *Ornithoctonus huwena*. PLoS ONE.

[38] He Q, Duan Z, Yu Y, Liu Z, Liang S (2013). The venom gland transcriptome of *Latrodectus tredecimguttatus* revealed by deep sequencing and cDNA library analysis. PLoS ONE.

[39] Ikeda N (2010). Unique structural characteristics and evolution of a cluster of venom phospholipase A2 isozyme genes of *Protobothrops flavoviridis* snake. Gene.

[40] Undheim EA (2015). Weaponization of a hormone: convergent recruitment of hyperglycemic hormone into the venom of arthropod predators. Structure.

[41] Michálek O, Řezáč M, Líznarová E, Symondson WOC, Pekár S (2018). Silk versus venom: alternative capture strategies employed by closely related myrmecophagous spiders. Biol. J. Lin. Soc..

[42] Pekar S (2018). Venom gland size and venom complexity-essential trophic adaptations of venomous predators: a case study using spiders. Mol. Ecol..

[43] Chen J (2008). Transcriptome analysis revealed novel possible venom components and cellular processes of the tarantula *Chilobrachys jingzhao* venom gland. Toxicon.

[44] Binford GJ (2009). Molecular evolution, functional variation, and proposed nomenclature of the gene family that includes sphingomyelinase D in sicariid spider venoms. Mol. Biol. Evol..

[45] Chou HH, Holmes MH (2001). DNA sequence quality trimming and vector removal. Bioinformatics.

[46] Sorek R, Safer HM (2003). A novel algorithm for computational identification of contaminated EST libraries. Nucl. Acids Res..

[47] Nishikawa T (2002). Database and analysis system for cDNA clones obtained from full-length enriched cDNA libraries. Silico Biol..

[48] Petersen TN, Brunak S, von Heijne G, Nielsen H (2011). SignalP 4.0: discriminating signal peptides from transmembrane regions. Nat. Methods.

[49] Wong ES, Hardy MC, Wood D, Bailey T, King GF (2013). SVM-based prediction of propeptide cleavage sites in spider toxins identifies toxin innovation in an Australian tarantula. PLoS ONE.

[50] Gracy J (2008). KNOTTIN: the knottin or inhibitor cystine knot scaffold in 2007. Nucl. Acids Res...

[51] Postic G, Gracy J, Perin C, Chiche L, Gelly JC (2018). KNOTTIN: the database of inhibitor cystine knot scaffold after 10 years, toward a systematic structure modeling. Nucl. Acids Res..

[52] Larkin MA (2007). Clustal W and Clustal X version 2.0. Bioinformatics.

[53] Kumar S, Stecher G, Tamura K (2016). MEGA7: molecular evolutionary genetics analysis version 7.0 for bigger datasets. Mol. Biol. Evol..

[54] Kumar S, Nei M, Dudley J, Tamura K (2008). MEGA: a biologist-centric software for evolutionary analysis of DNA and protein sequences. Brief Bioinform..

[55] Tamura K (2011). MEGA5: molecular evolutionary genetics analysis using maximum likelihood, evolutionary distance, and maximum parsimony methods. Mol. Biol. Evol..

