# Supplementary material for: Molecular diversity and evolutionary trends of cysteine-rich peptides from the venom glands of Chinese spider Heteropoda venatoria
Source: Sci Rep. 2021 Feb 5;11:3211. doi: 10.1038/s41598-021-82668-5 (PMC7865051; doi:10.1038/s41598-021-82668-5)
Supplement: Supplementary file 1 — Supplementary Information 1. [file 41598_2021_82668_MOESM1_ESM.docx]

**Molecular diversity** **and evolutionary trends of cysteine-rich peptides from the venom glands of Chinese spider** ***Heteropoda venatoria***

**Short running title: Diversity and evolution of** ***Heteropoda venatoria* toxins**

Jie Luo^a,†^, Yiying Ding^a,†^, Zhihao Peng^a^, Kezhi Chen^a^, Xuewen Zhang^a^, Tiaoyi Xiao^b^, Jinjun Chen^a,c^*

^a^College of Bioscience and Biotechnology, Hunan Agricultural University, Changsha 410128, P.R. China;

^b^College of Animal Science and Technology, Hunan Agricultural University, Changsha 410128, P.R. China;

^c^Hunan Provincial Engineering Technology Research Center for Cell Mechanics and Function Analysis, Changsha 410128, P.R. China

*Corresponding author: [chhncjj@126.com](mailto:chhncjj@126.com) (Jinjun Chen)

^†^ Jie Luo and Yiying Ding contributed equally to this work.

**Supplementary Information**

**
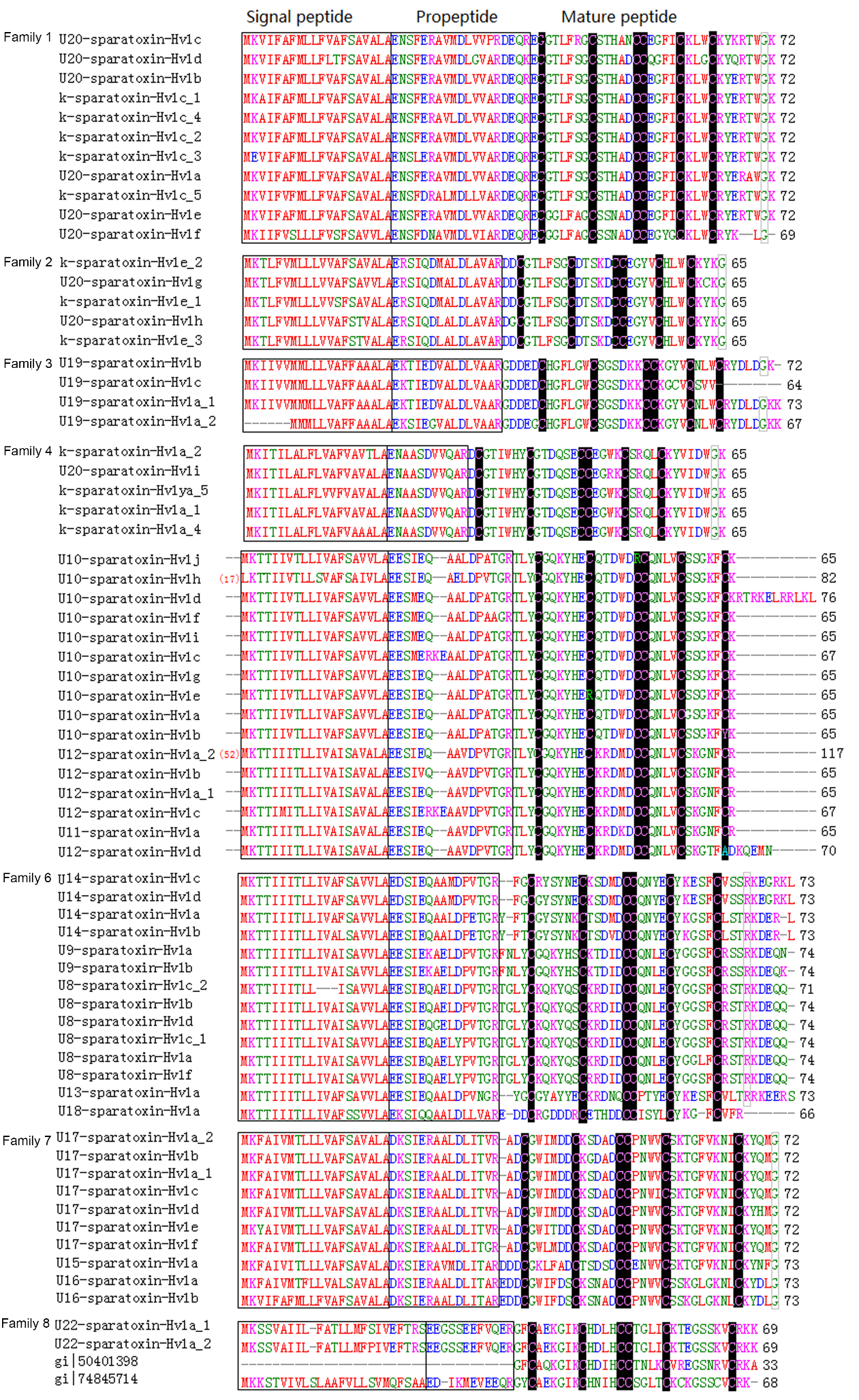
**

**Suppl-Fig. 1** Homology alignment of Family 1-8 ICKs with 6-cys motif. The putative signal peptides and propeptides are indicated by dark box. C-terminally, prosequences are indicated by gray box. Conserved cysteine residues are indicated by filled with black.

**
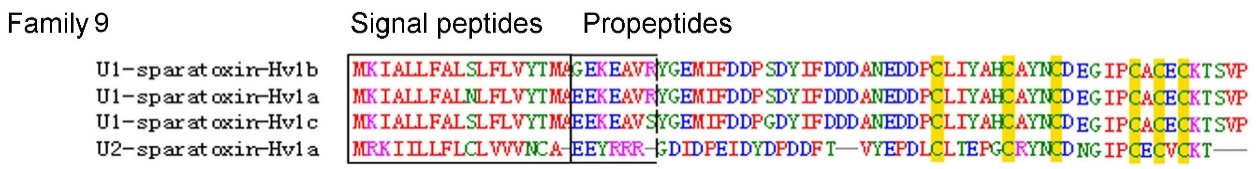
**

**Suppl-Fig. 2** Multiple sequence alignment of Family 9 precursors and amino acid sequences from *H. venatoria*. The putative signal peptides and propeptides are boxed and cysteines of mature peptide are in gray shadow. Gaps (dashes) were introduced to maximize the deduced polypeptide sequence similarities.

**
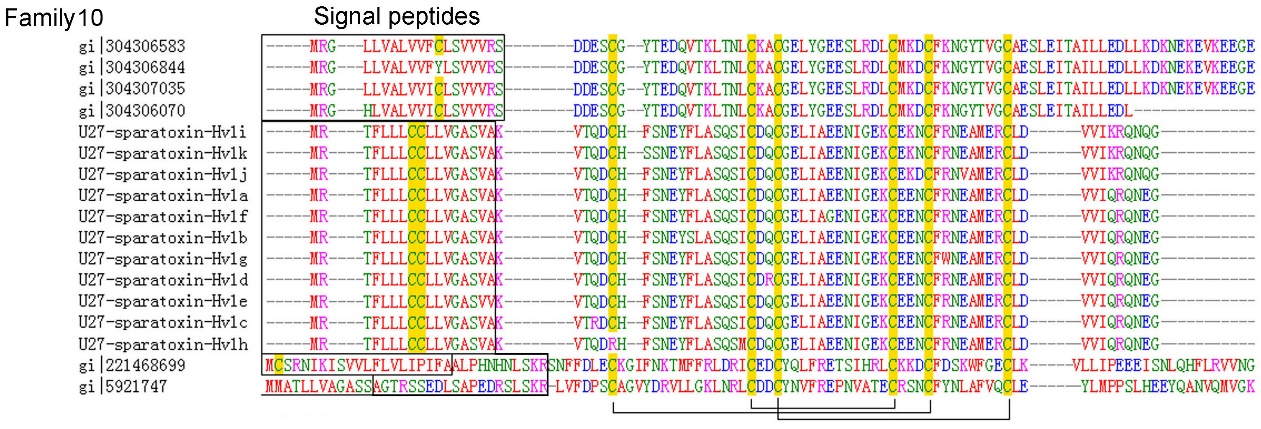
**

**Suppl-Fig. 3** Multiple sequence alignment of Family 10 precursors and amino acid sequences from *H. venatoria*. The putative signal peptides and propeptides are boxed and cysteines of mature peptide are in gray shadow. Gaps (dashes) were introduced to maximize the deduced polypeptide sequence similarities.

**
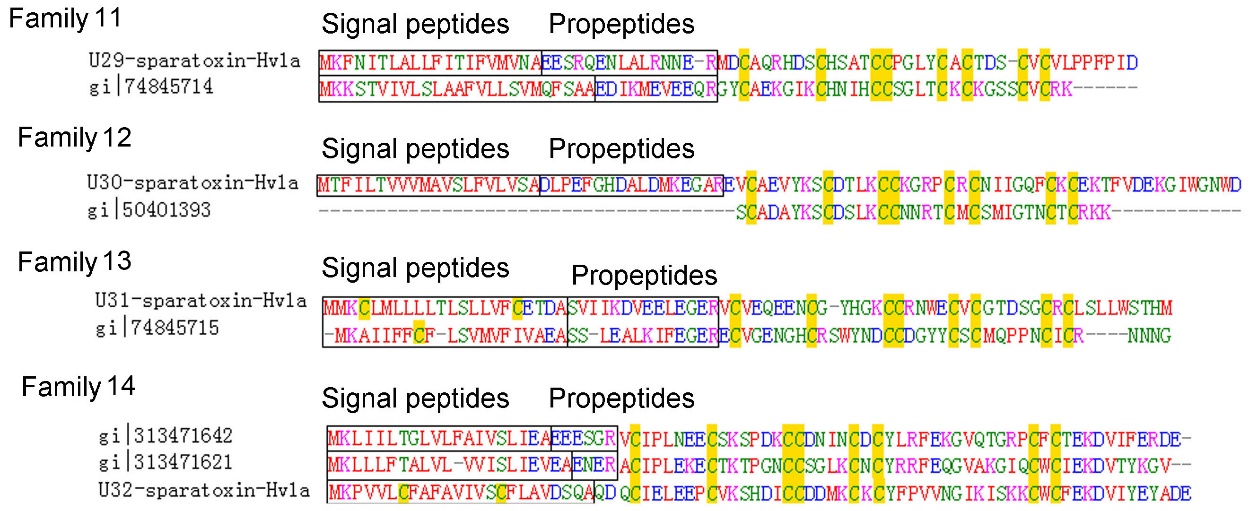
**

**Suppl-Fig. 4** Multiple sequence alignment of Family 11-14 precursors and amino acid sequences from *H. venatoria*. The putative signal peptides and propeptides are boxed and cysteines of mature peptide are in gray shadow. Gaps (dashes) were introduced to maximize the deduced polypeptide sequence similarities.

**
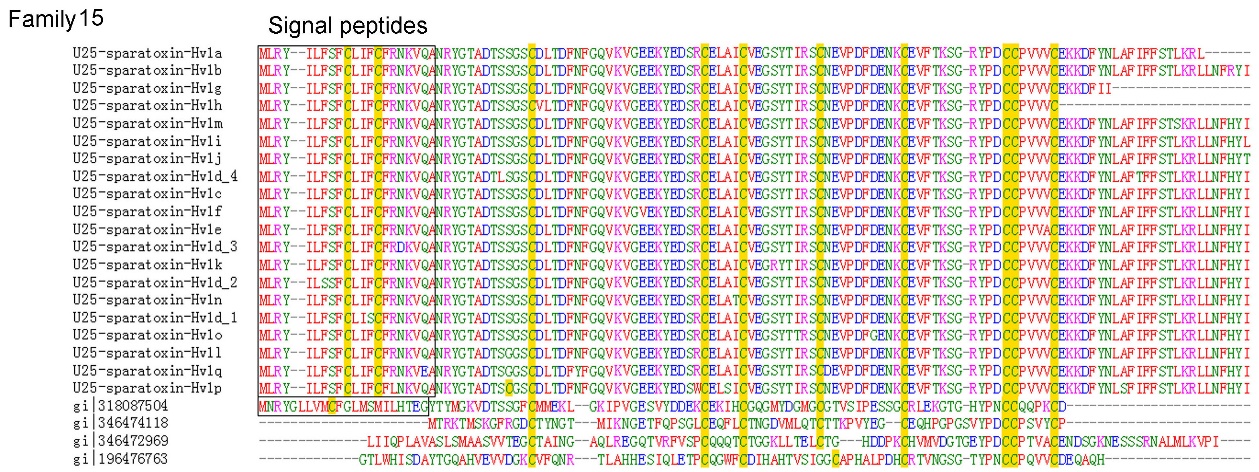
**

**Suppl-Fig. 5** Multiple sequence alignment of Family 15 precursors and amino acid sequences from *H. venatoria*. The putative signal peptides and propeptides are boxed and cysteines of mature peptide are in gray shadow. Gaps (dashes) were introduced to maximize the deduced polypeptide sequence similarities.

**
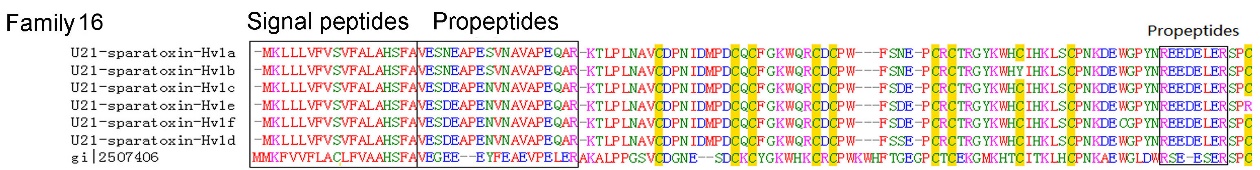
**

**Suppl-Fig. 6** Multiple sequence alignment of Family 16 precursors and amino acid sequences from *H. venatoria*. The putative signal peptides and propeptides are boxed and cysteines of mature peptide are in gray shadow. Gaps (dashes) were introduced to maximize the deduced polypeptide sequence similarities.


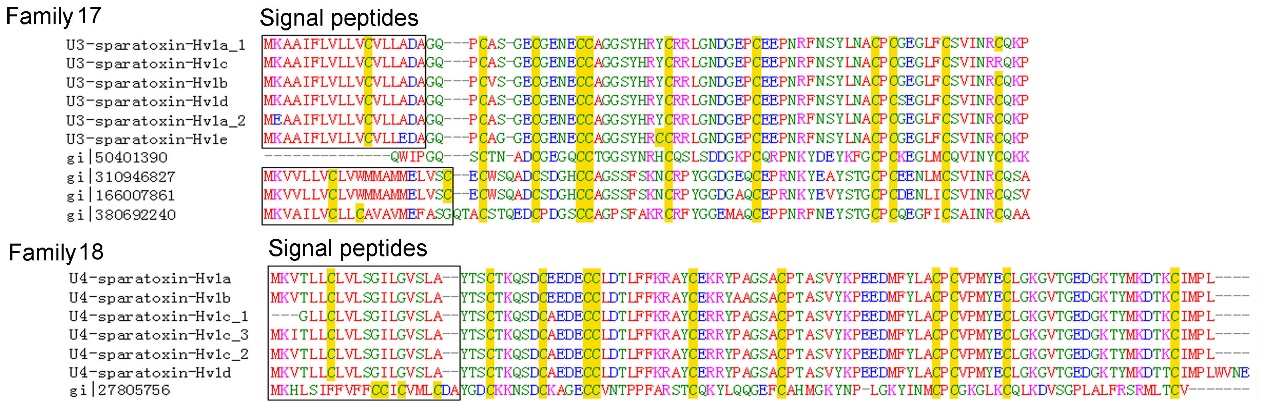


**Suppl-Fig. 7** Multiple sequence alignment of Family 17 and 18 precursors and amino acid sequences from *H. venatoria*. The putative signal peptides and propeptides are boxed and cysteines of mature peptide are in gray shadow. Gaps (dashes) were introduced to maximize the deduced polypeptide sequence similarities.

**
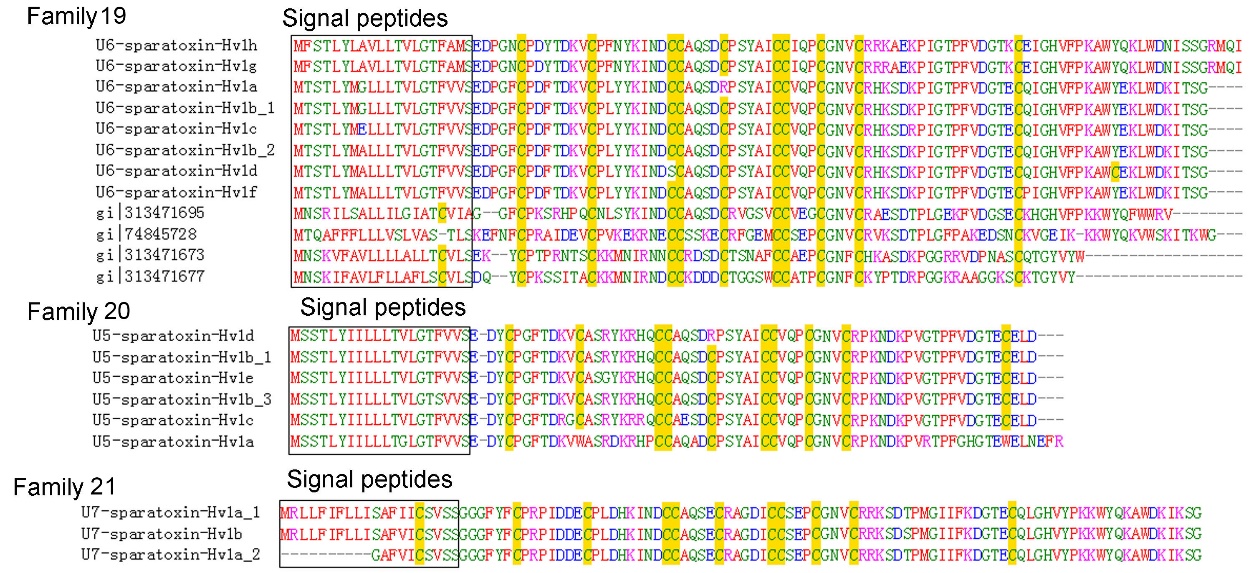
**

**Suppl-Fig. 8** Multiple sequence alignment of Family 19, 20 and 21 precursors and amino acid sequences from *H. venatoria*. The putative signal peptides and propeptides are boxed and cysteines of mature peptide are in gray shadow. Gaps (dashes) were introduced to maximize the deduced polypeptide sequence similarities.

**
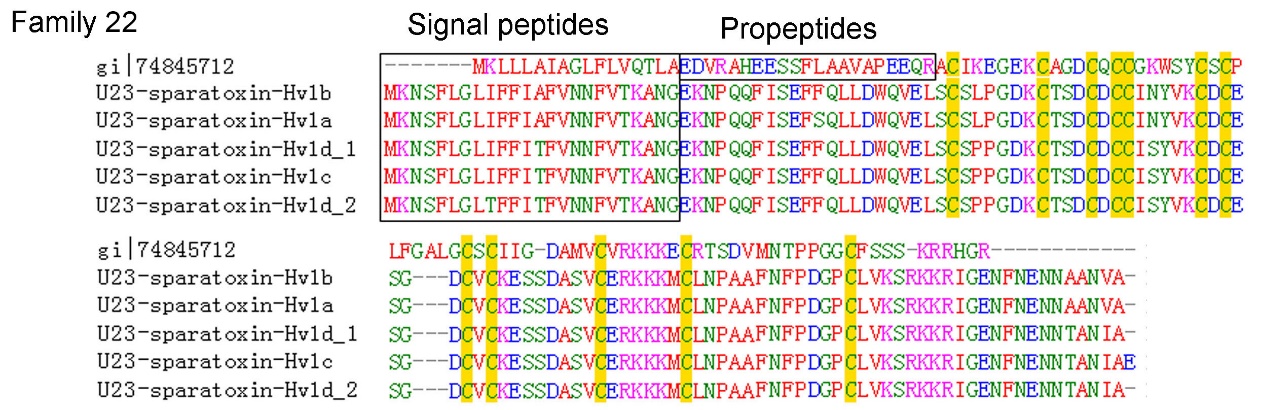
**

**Suppl-Fig. 9** Multiple sequence alignment of Family 22 precursors and amino acid sequences from *H. venatoria*. The putative signal peptides and propeptides are boxed and cysteines of mature peptide are in gray shadow. Gaps (dashes) were introduced to maximize the deduced polypeptide sequence similarities.

**
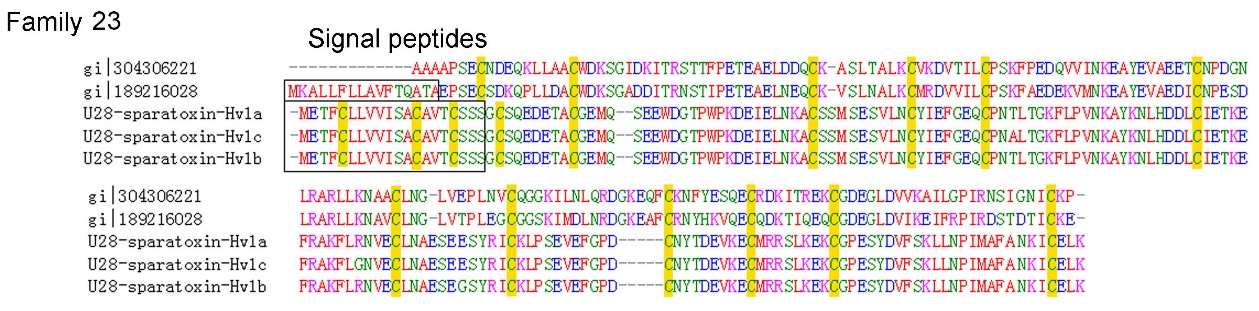
** **Suppl-Fig. 10** Multiple sequence alignment of Family 23 precursors and amino acid sequences from *H. venatoria*. The putative signal peptides are boxed and cysteines of mature peptide are in gray shadow. Gaps (dashes) were introduced to maximize the deduced polypeptide sequence similarities.

**
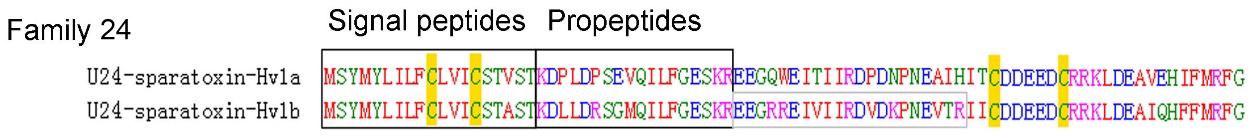
** **Suppl-Fig. 11** Multiple sequence alignment of Family 24 precursors and amino acid sequences from *H. venatoria*. The putative signal peptides and propeptides are boxed and cysteines of mature peptide are in gray shadow.
