# Supplementary material for: Molecular diversity and evolutionary trends of cysteine-rich peptides from the venom glands of Chinese spider Heteropoda venatoria
Source: Sci Rep. 2021 Feb 5;11:3211. doi: 10.1038/s41598-021-82668-5 (PMC7865051; doi:10.1038/s41598-021-82668-5)
Supplement: Supplementary file 3 — Supplementary Information 3. [file 41598_2021_82668_MOESM3_ESM.docx]

**Molecular diversity** **and evolutionary trends of cysteine-rich peptides from the venom glands of Chinese spider** ***Heteropoda venatoria***

**Short running title: Diversity and evolution of** ***Heteropoda venatoria* toxins**

Jie Luo^a,†^, Yiying Ding^a,†^, Zhihao Peng^a^, Kezhi Chen^a^, Xuewen Zhang^a^, Tiaoyi Xiao^b^, Jinjun Chen^a,c^*

^a^College of Bioscience and Biotechnology, Hunan Agricultural University, Changsha 410128, P.R. China;

^b^College of Animal Science and Technology, Hunan Agricultural University, Changsha 410128, P.R. China;

^c^Hunan Provincial Engineering Technology Research Center for Cell Mechanics and Function Analysis, Changsha 410128, P.R. China

*Corresponding author: [chhncjj@126.com](mailto:chhncjj@126.com) (Jinjun Chen)

^†^ Jie Luo and Yiying Ding contributed equally to this work.

Reversed Phase High Performance Liquid Chromatography


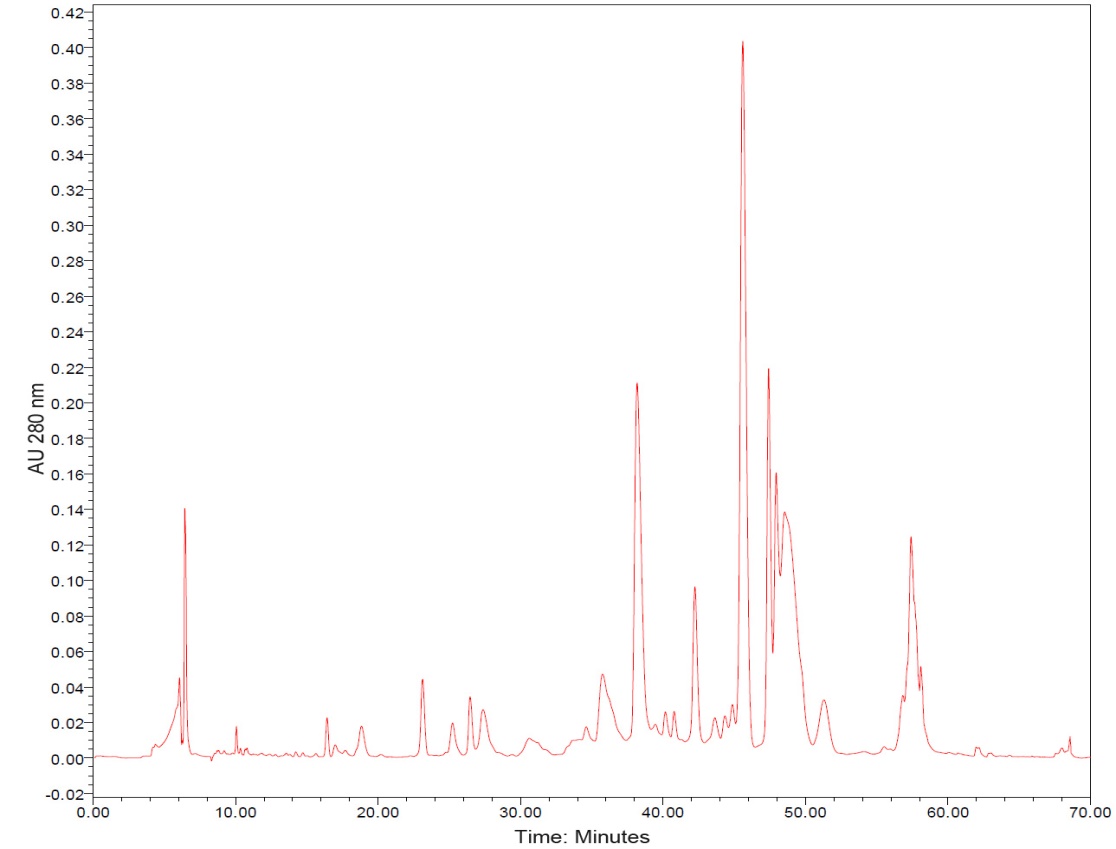


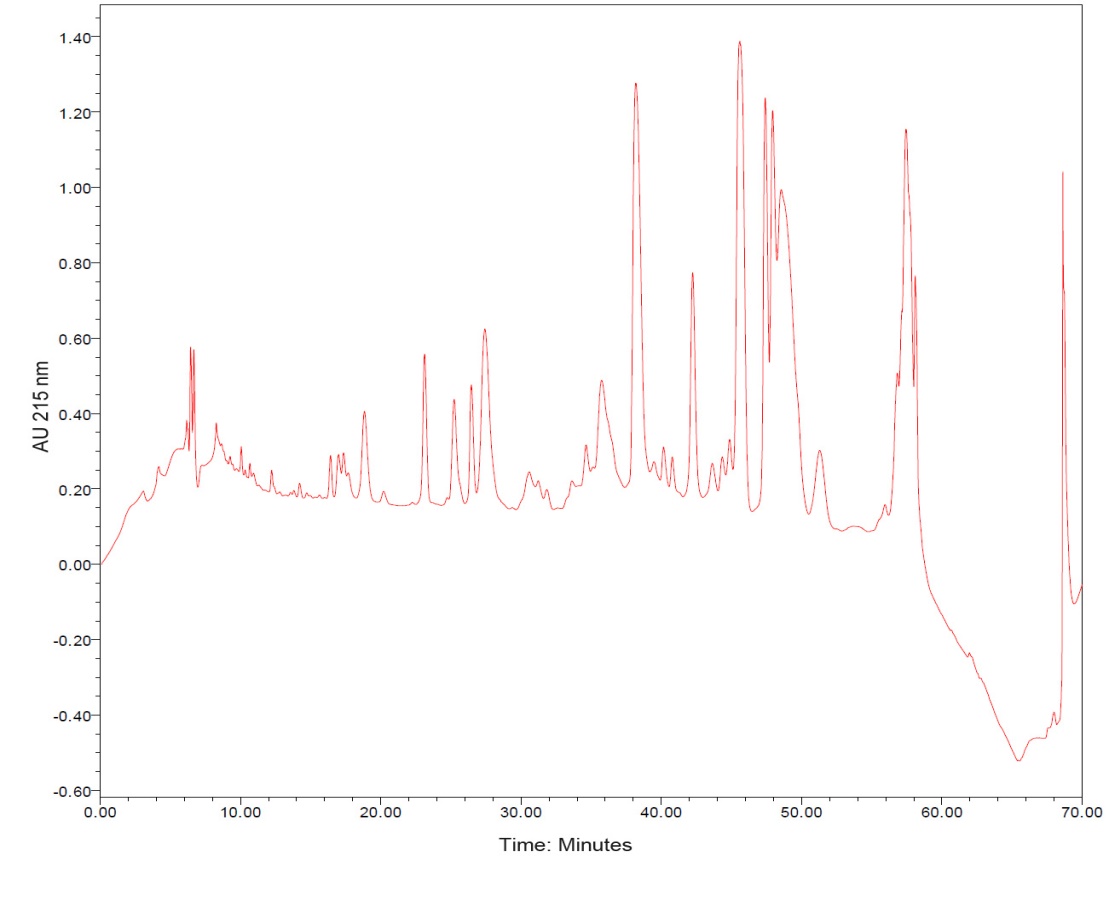


MALDI MS chromatogram


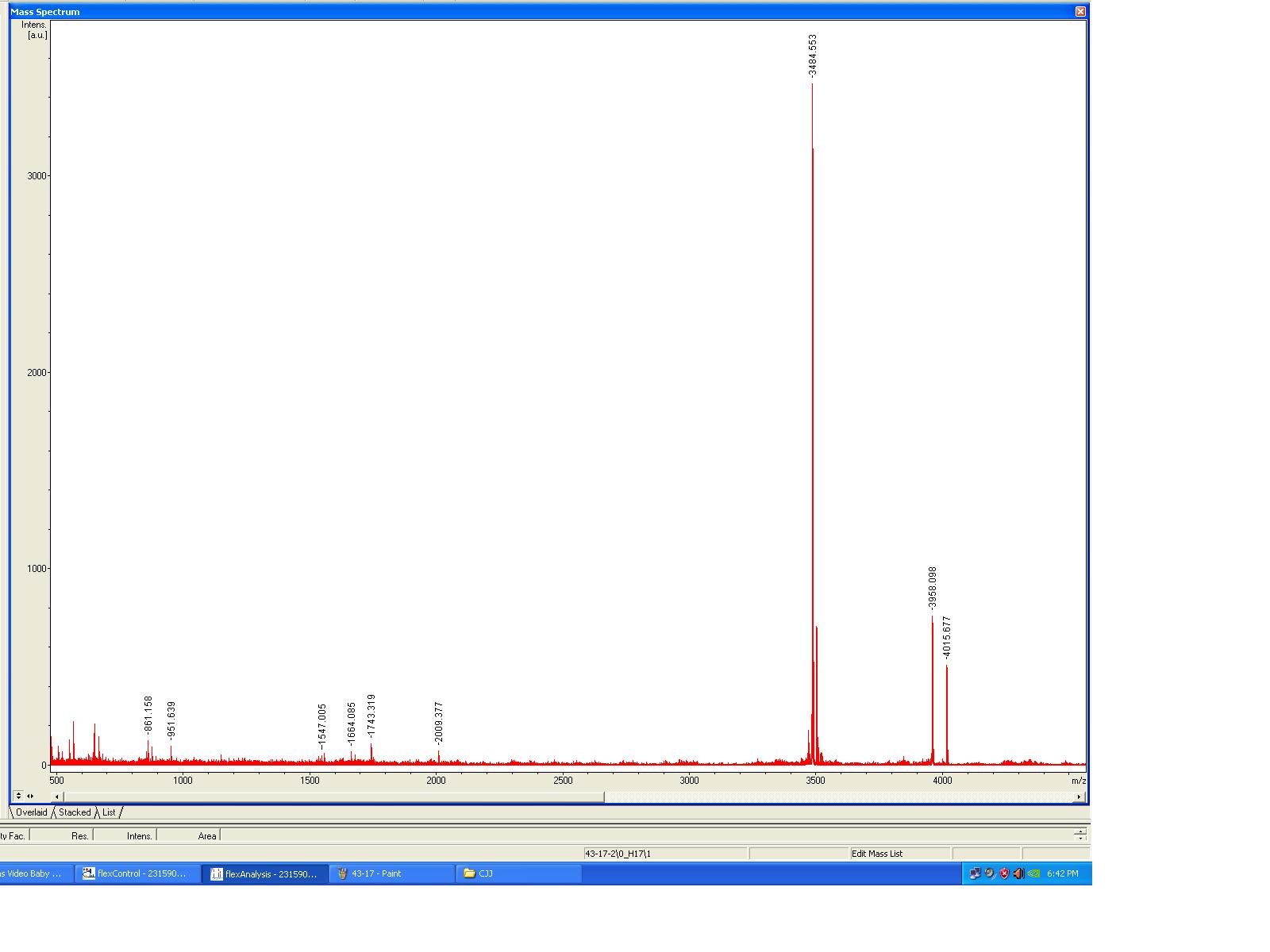


3485


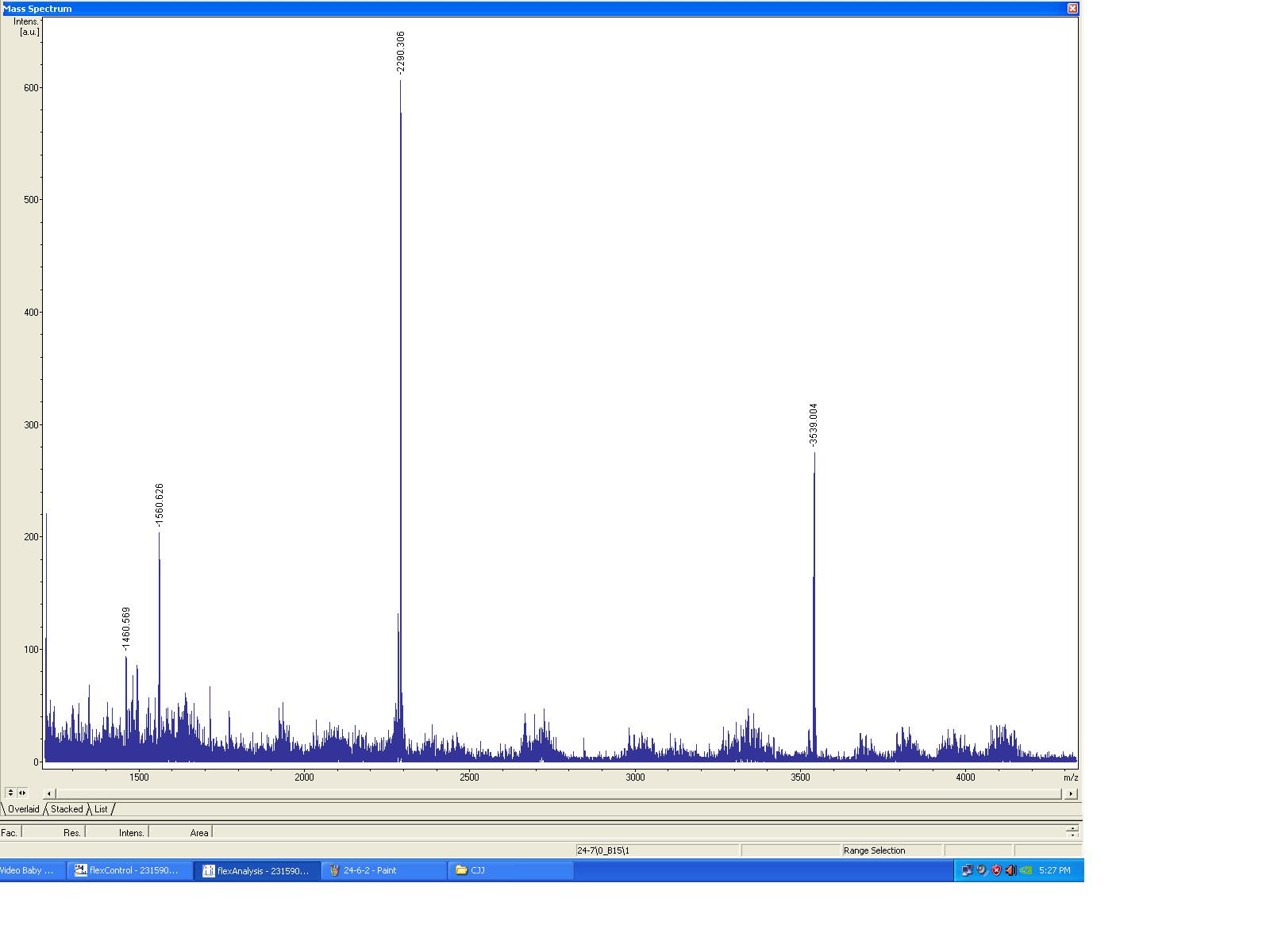


3539


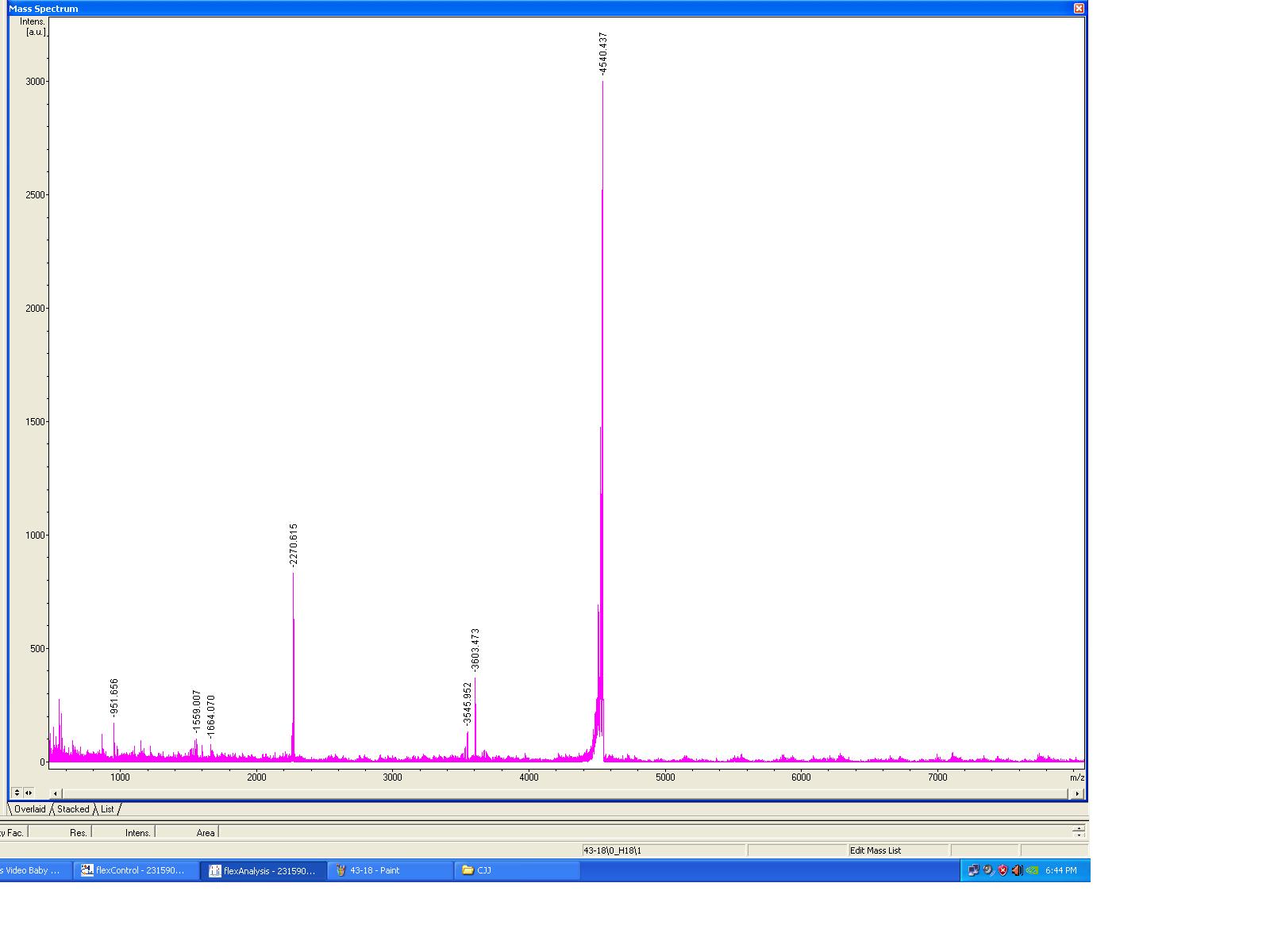


3545


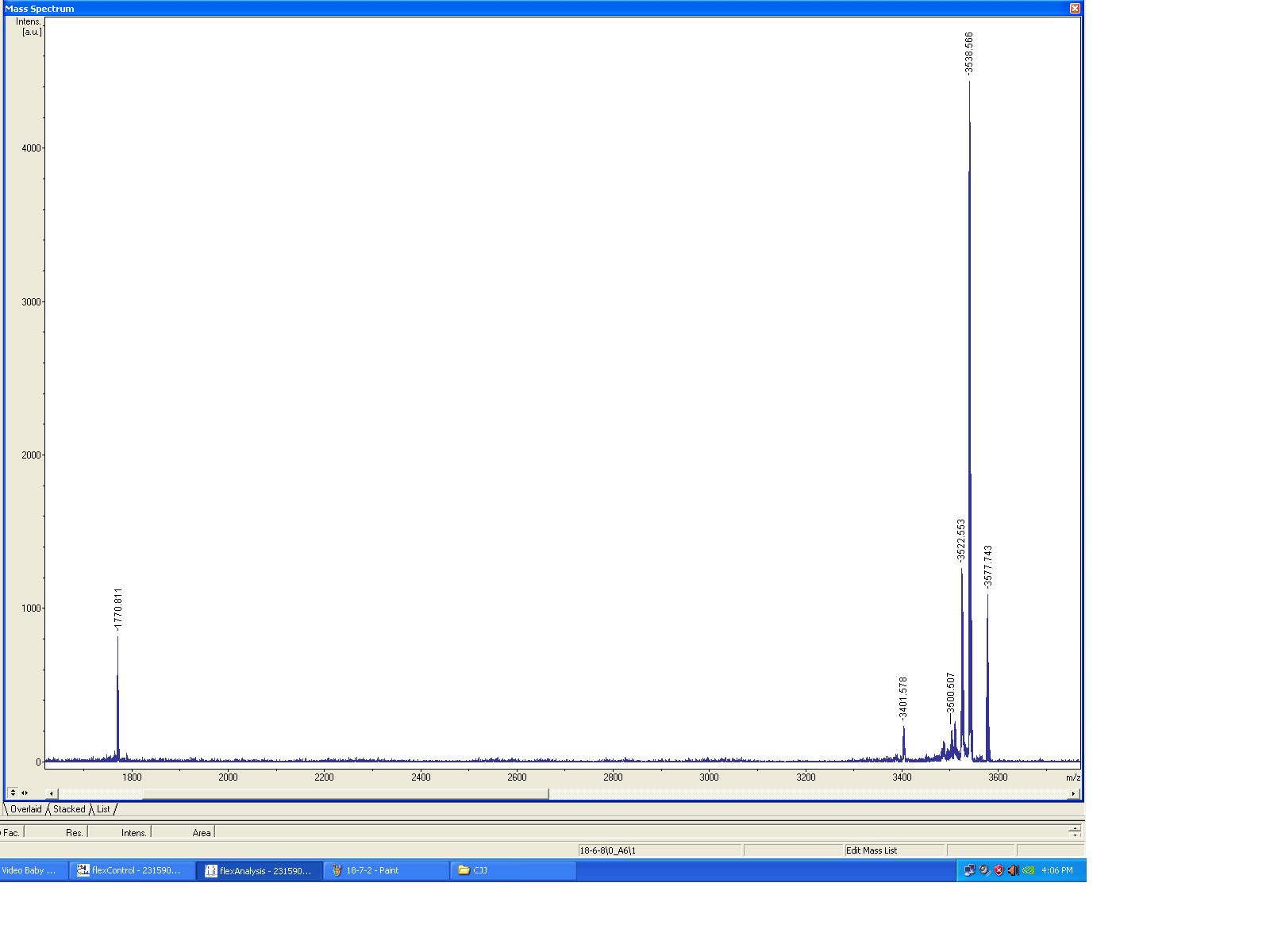


3539


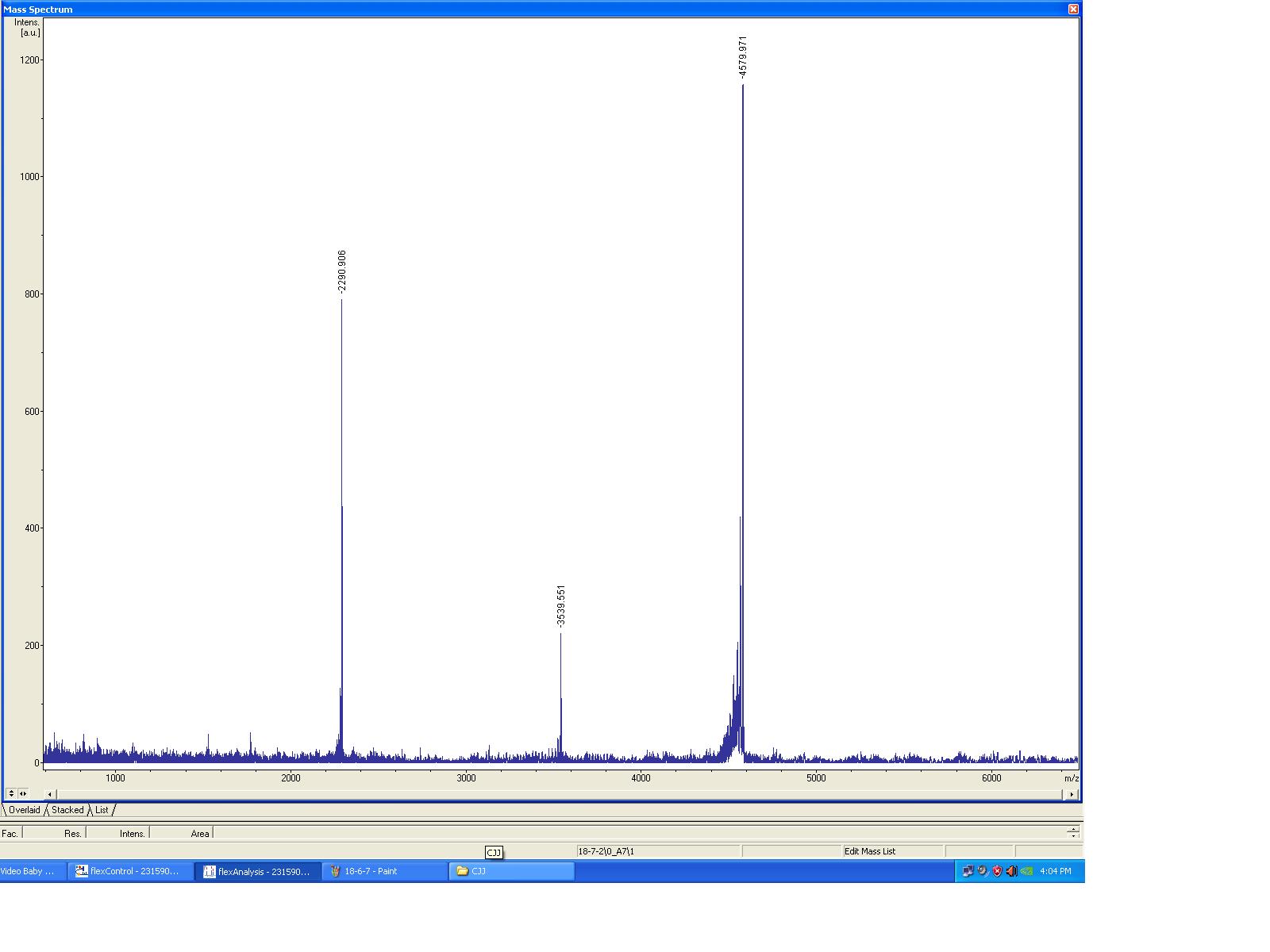


4580


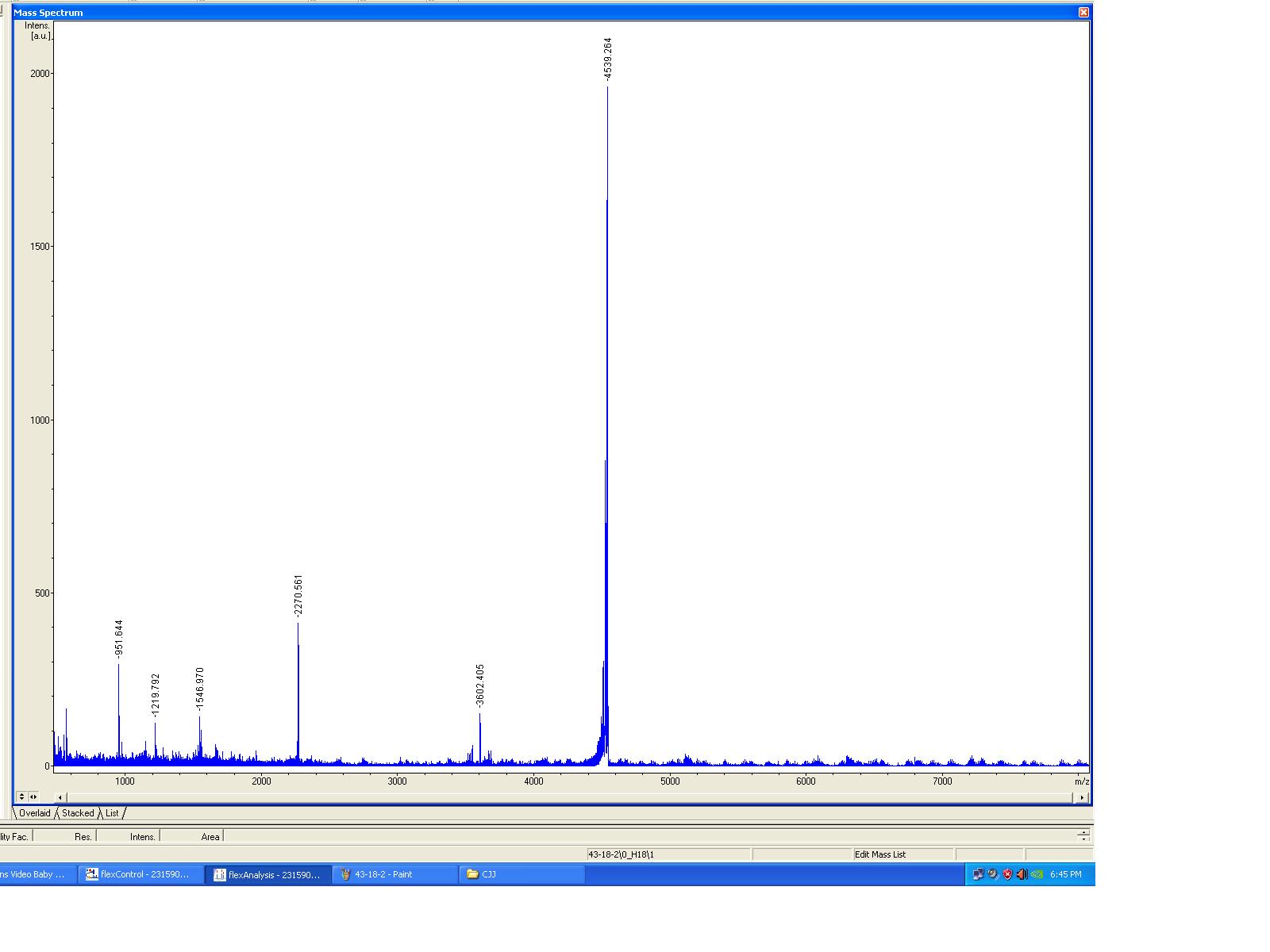


4539


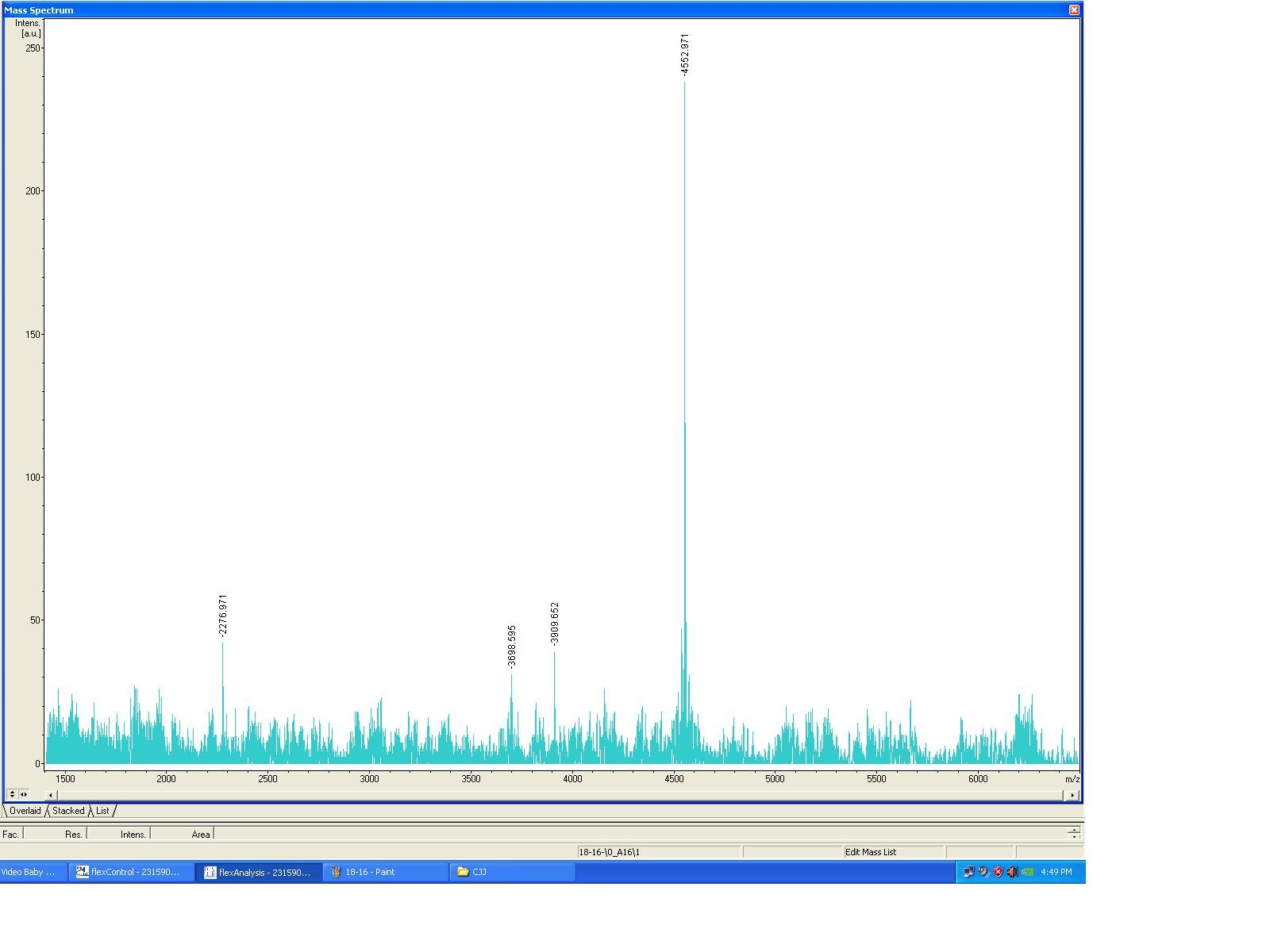


3698


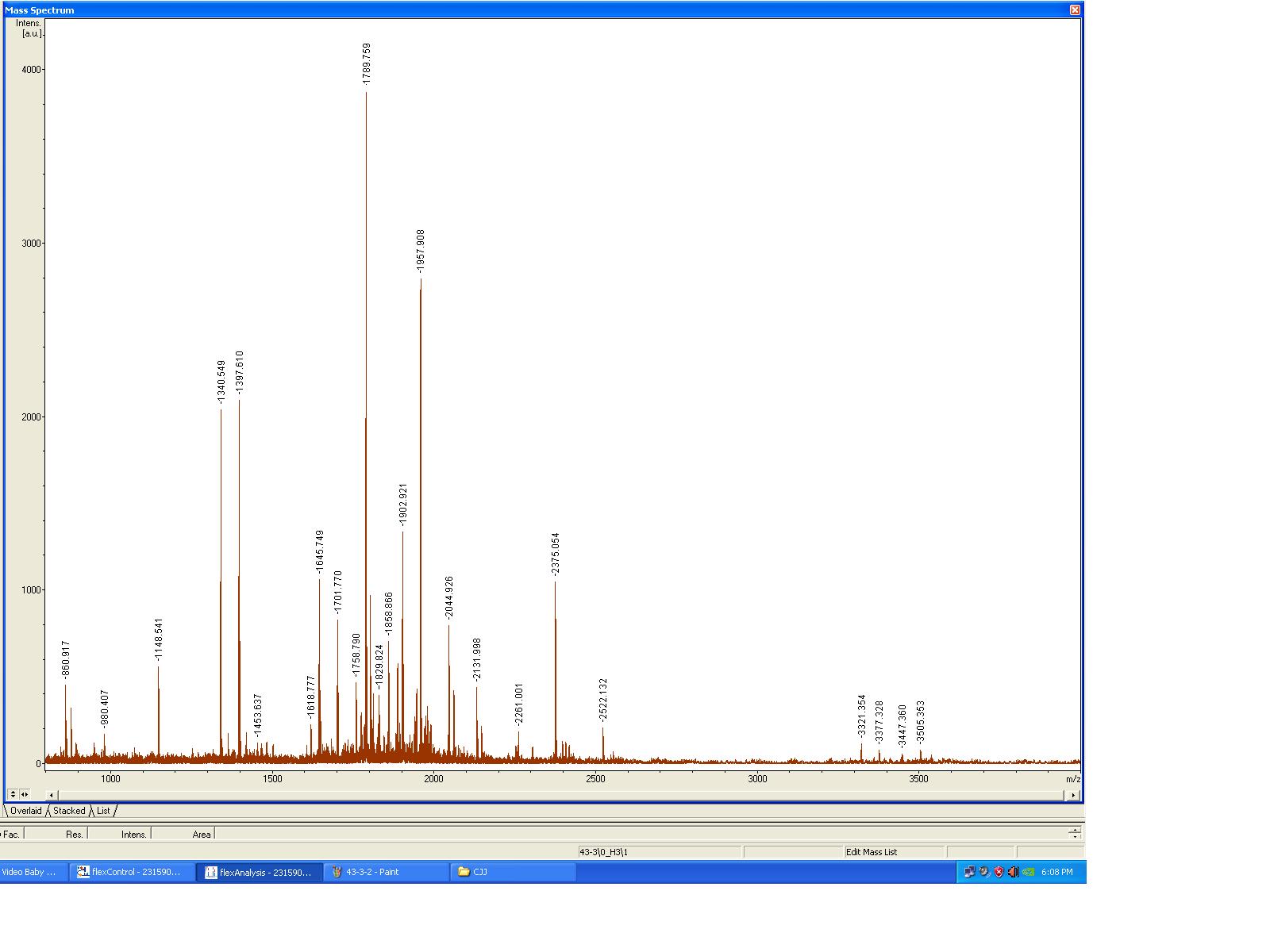


3377


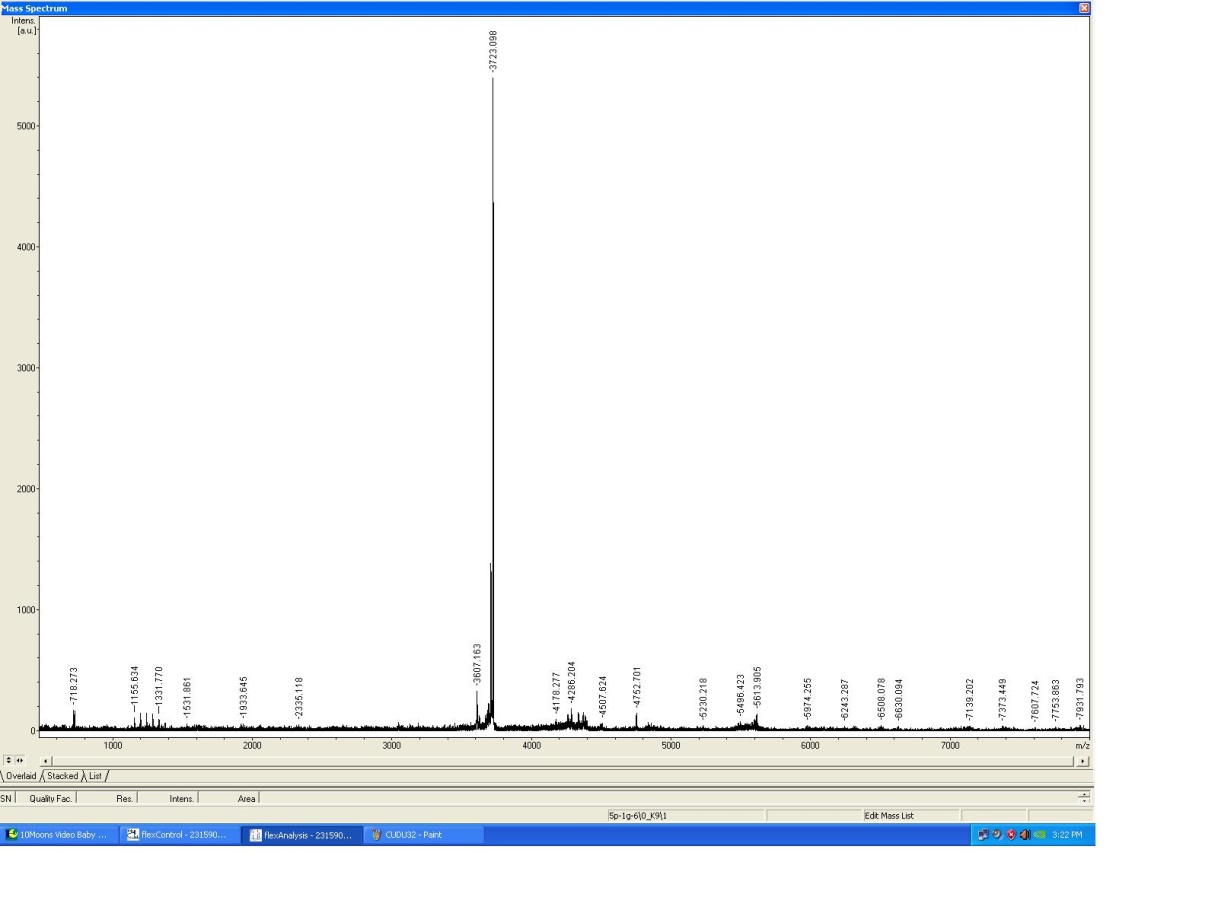


3723


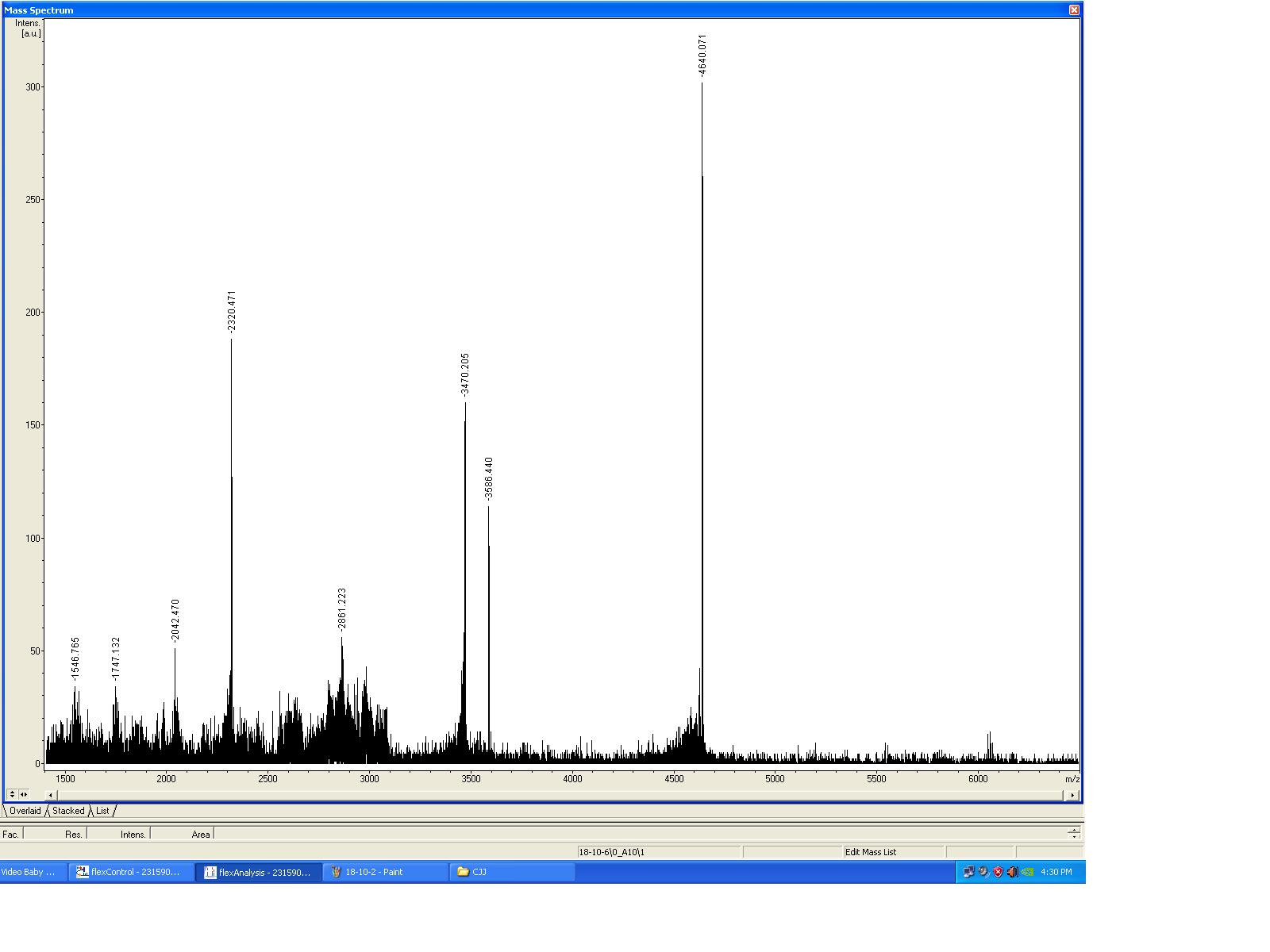


3586


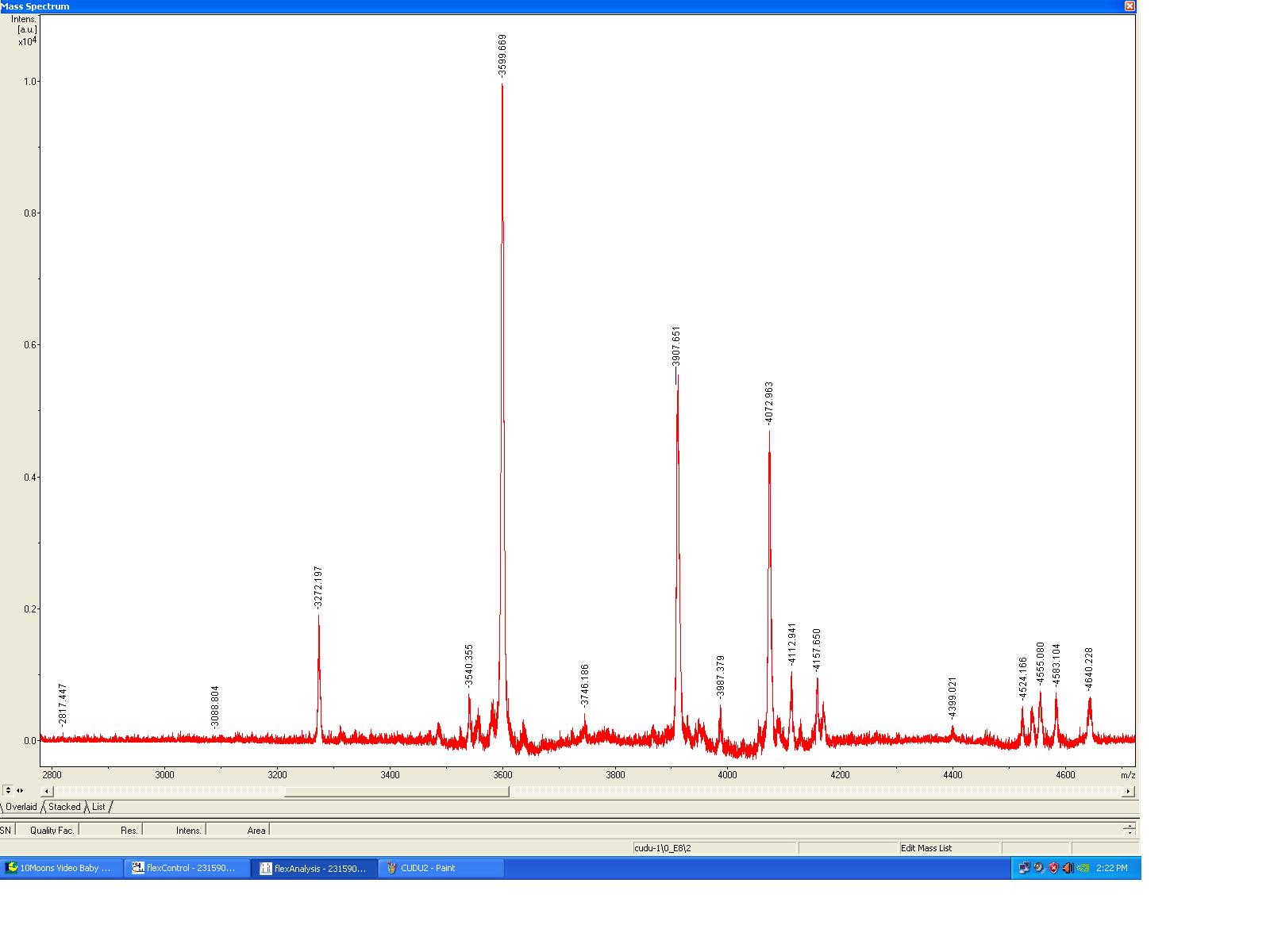


3599


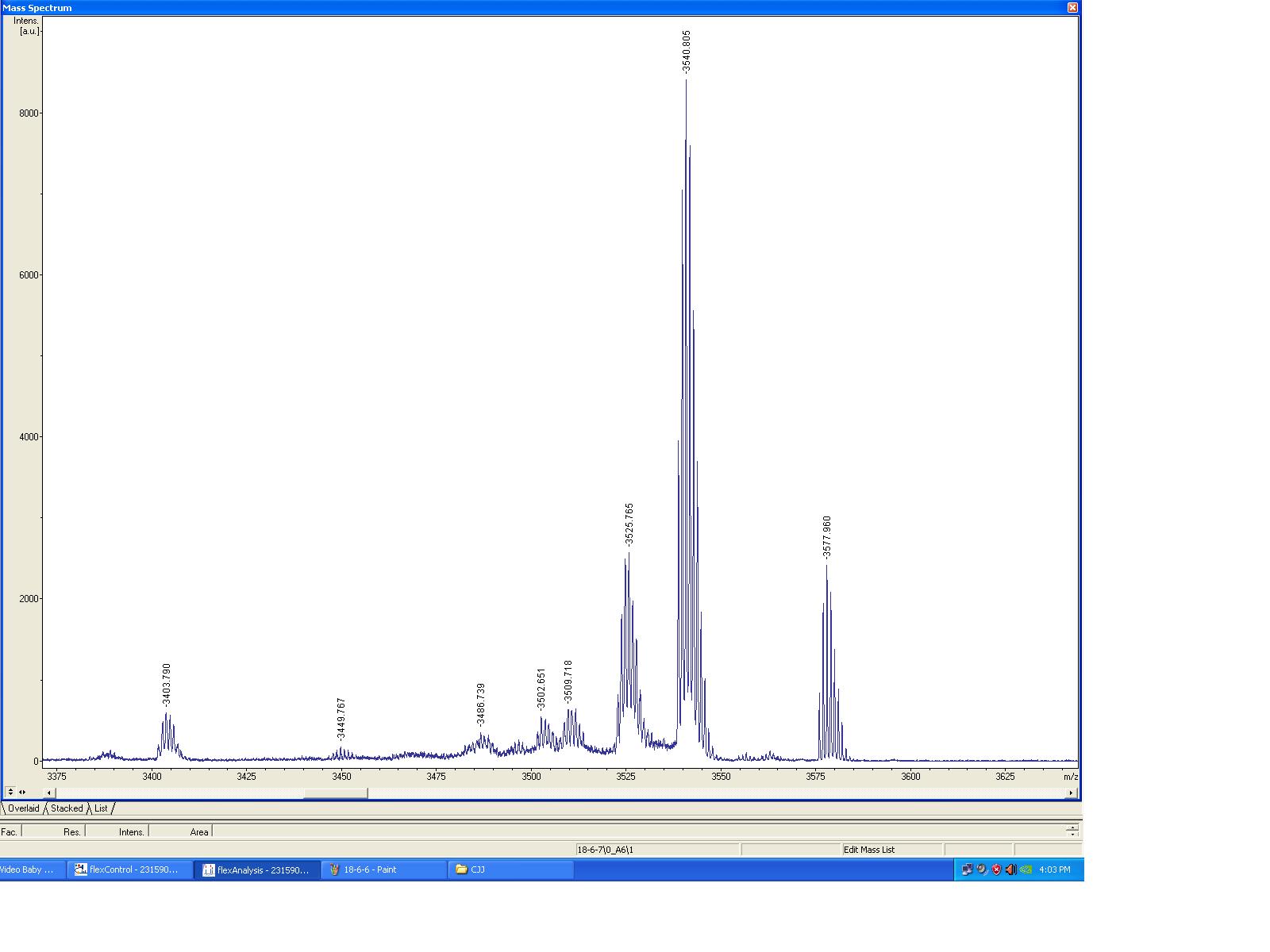


3502


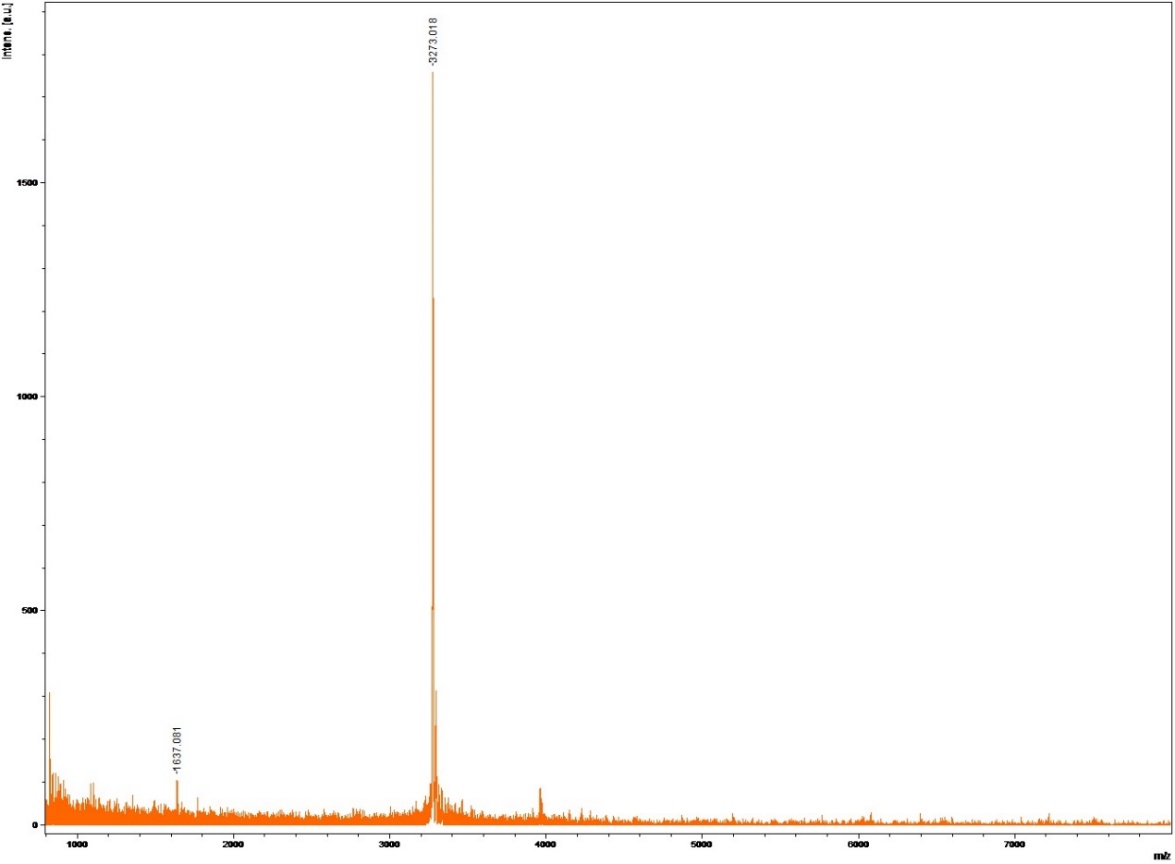


3273


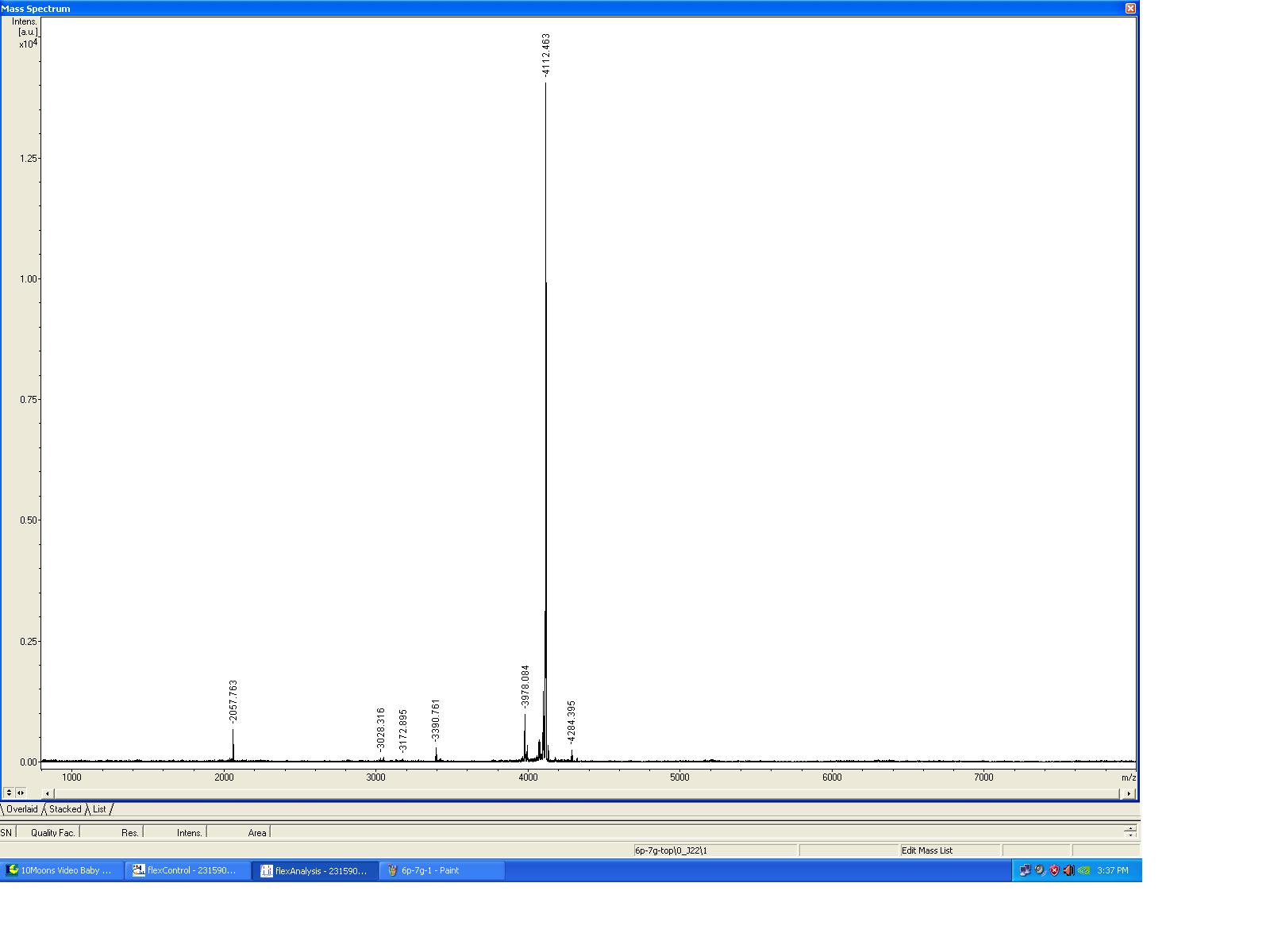


4112


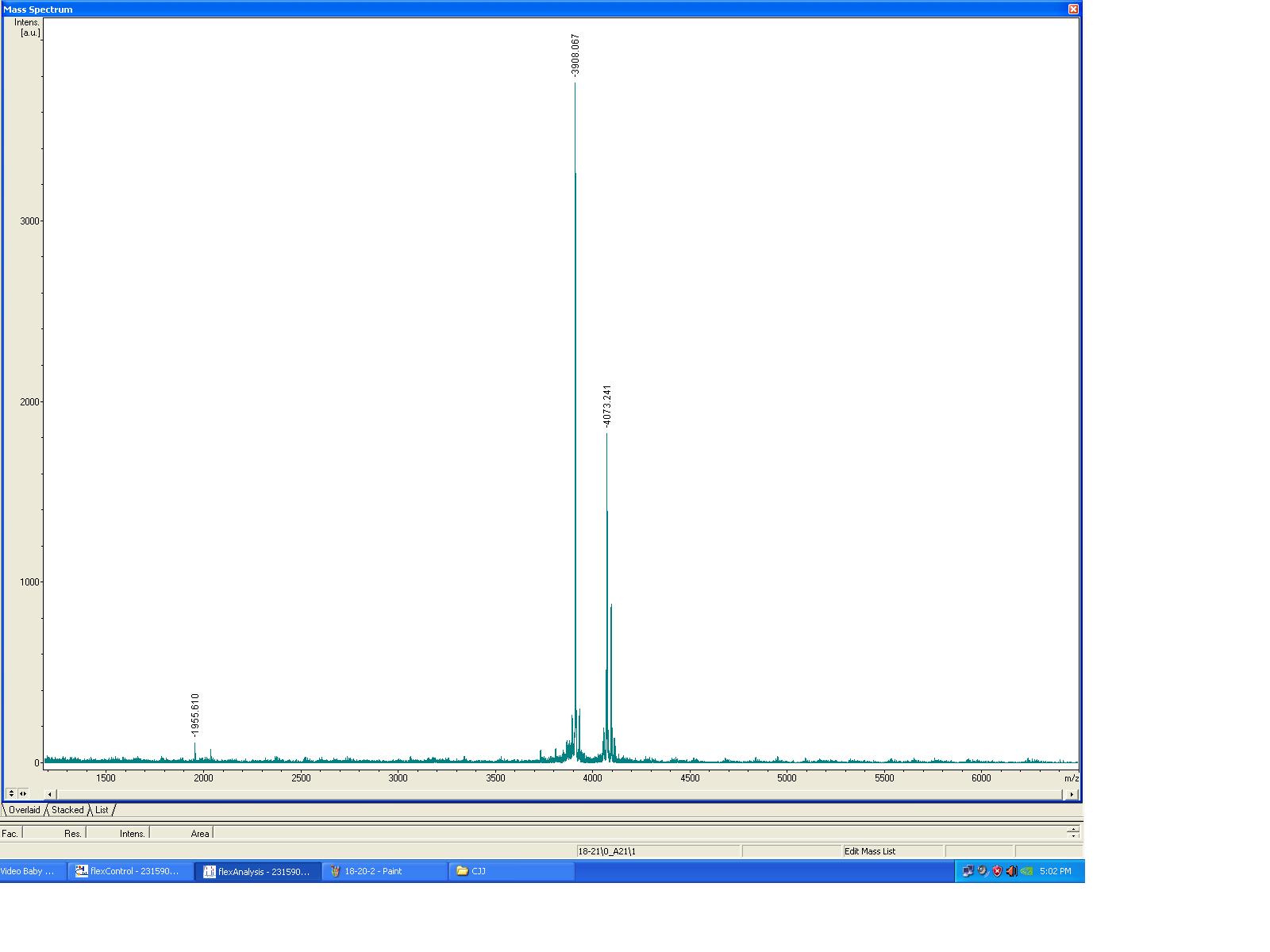


4073


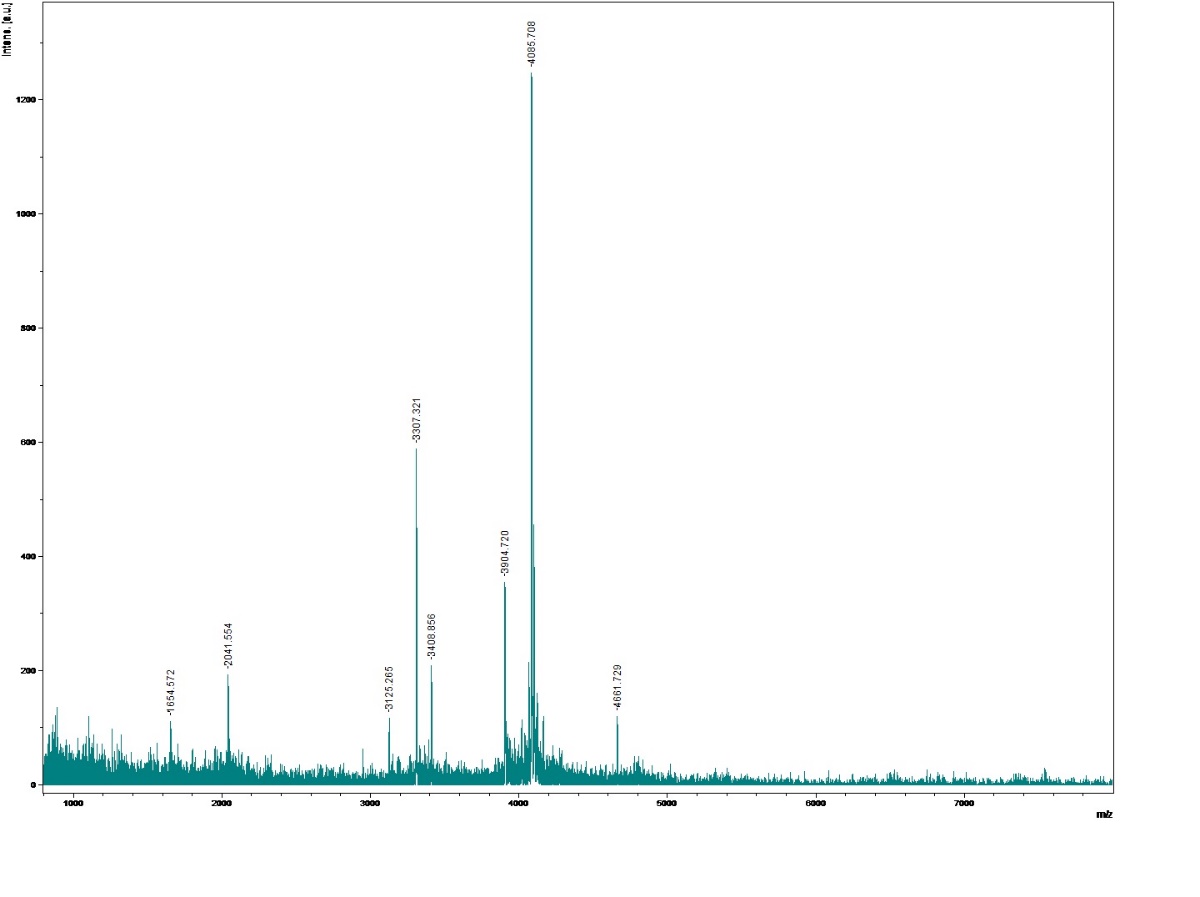


4085


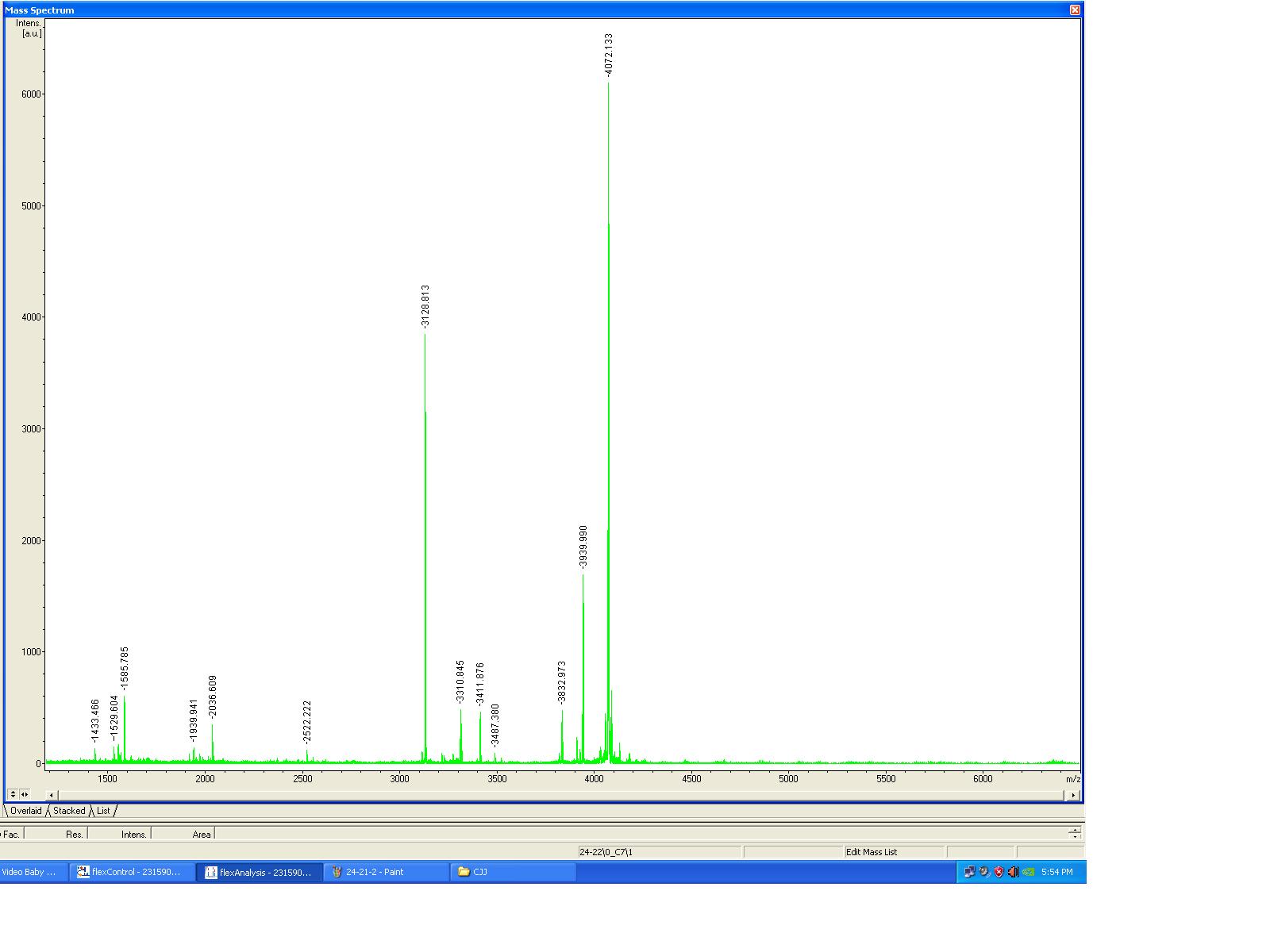


4072


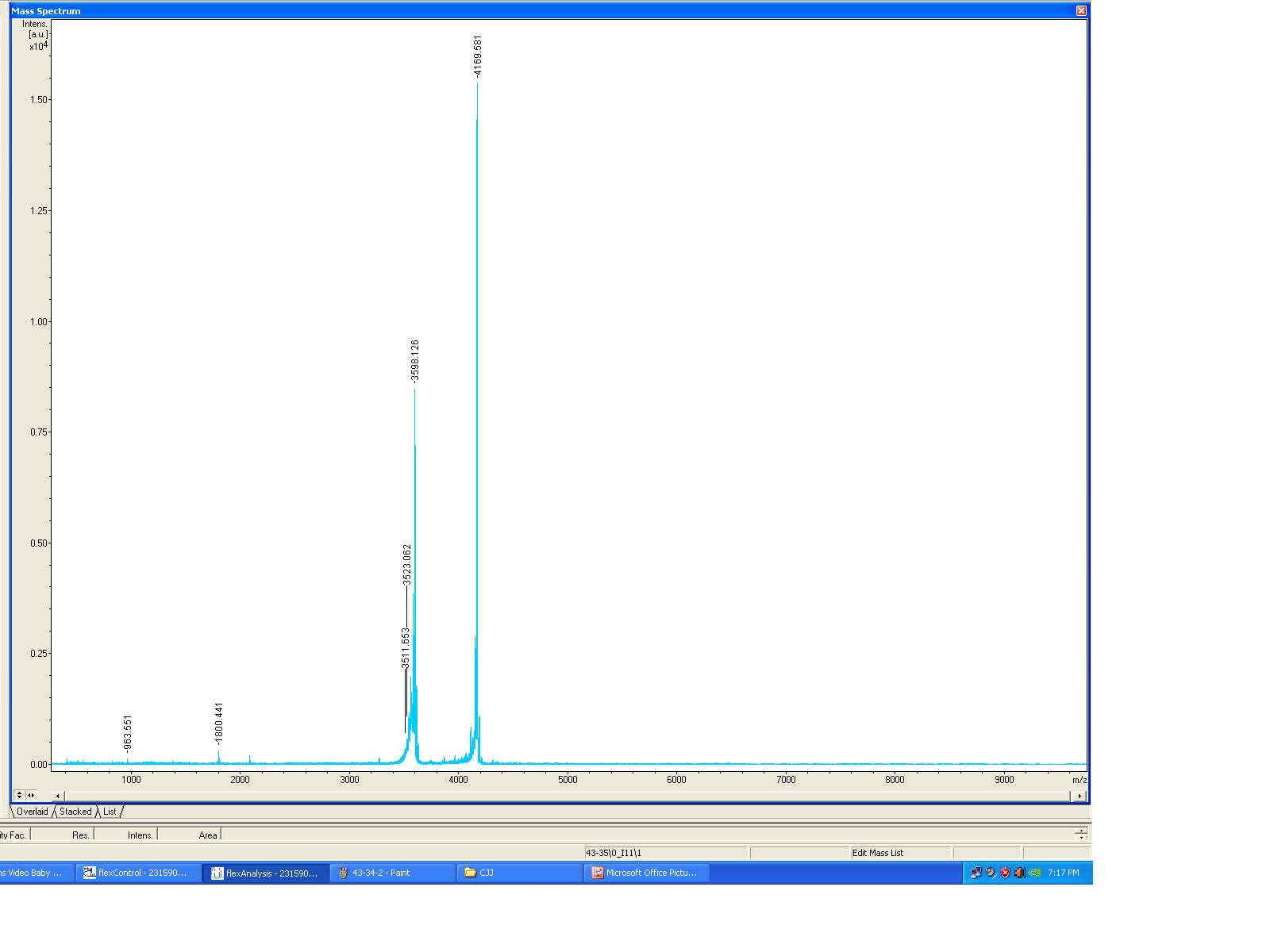


4169


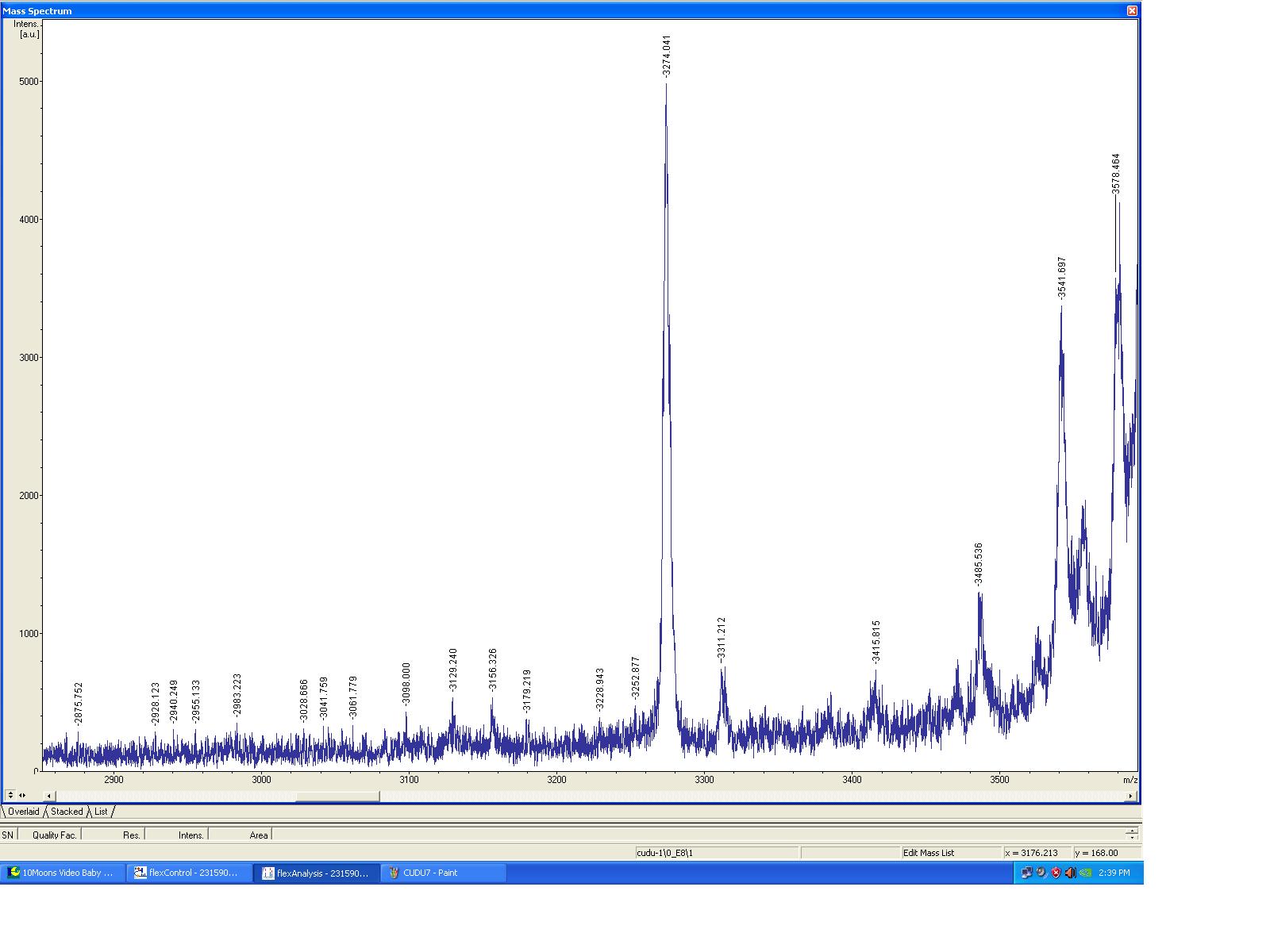


3578


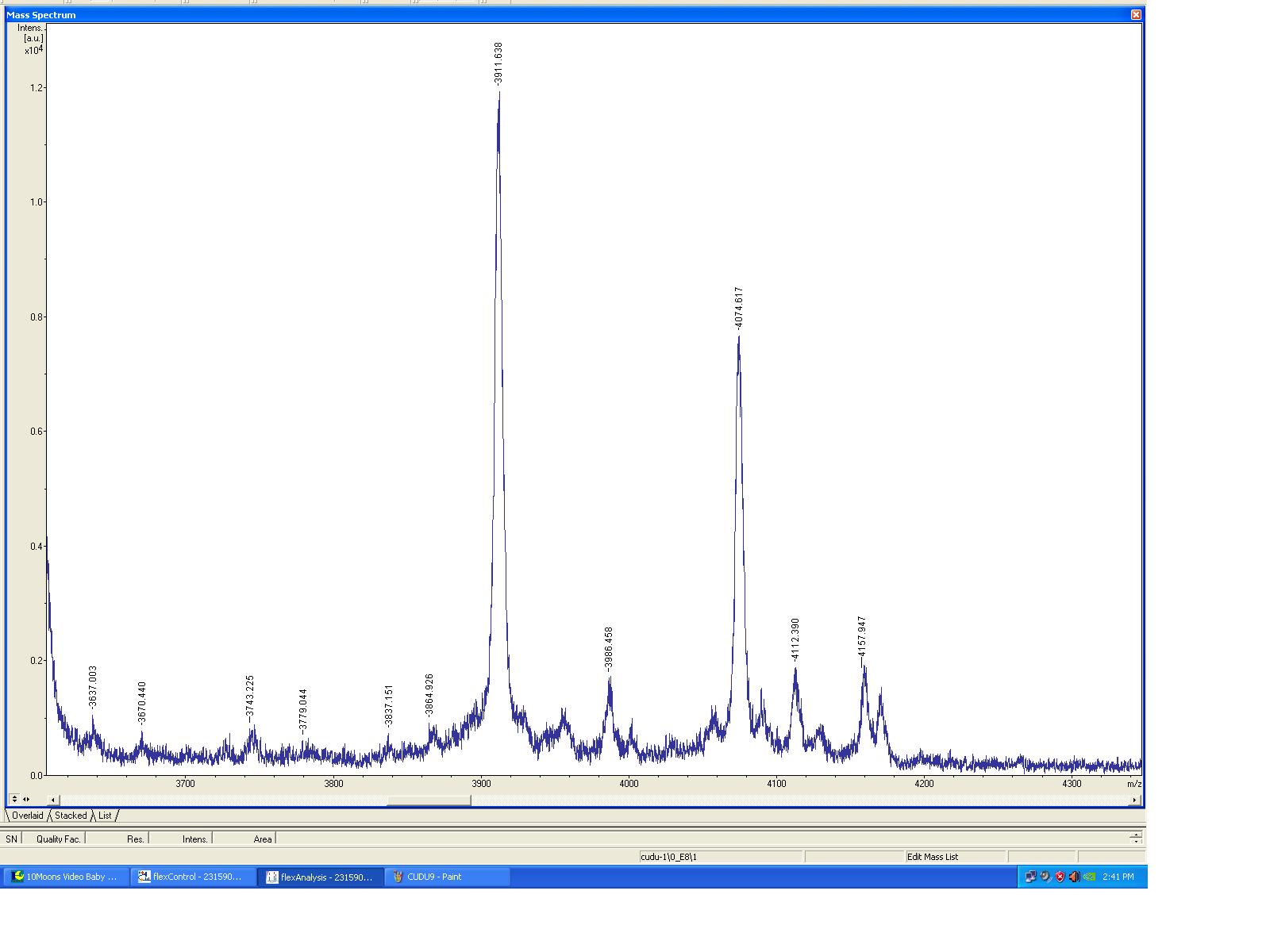


3986


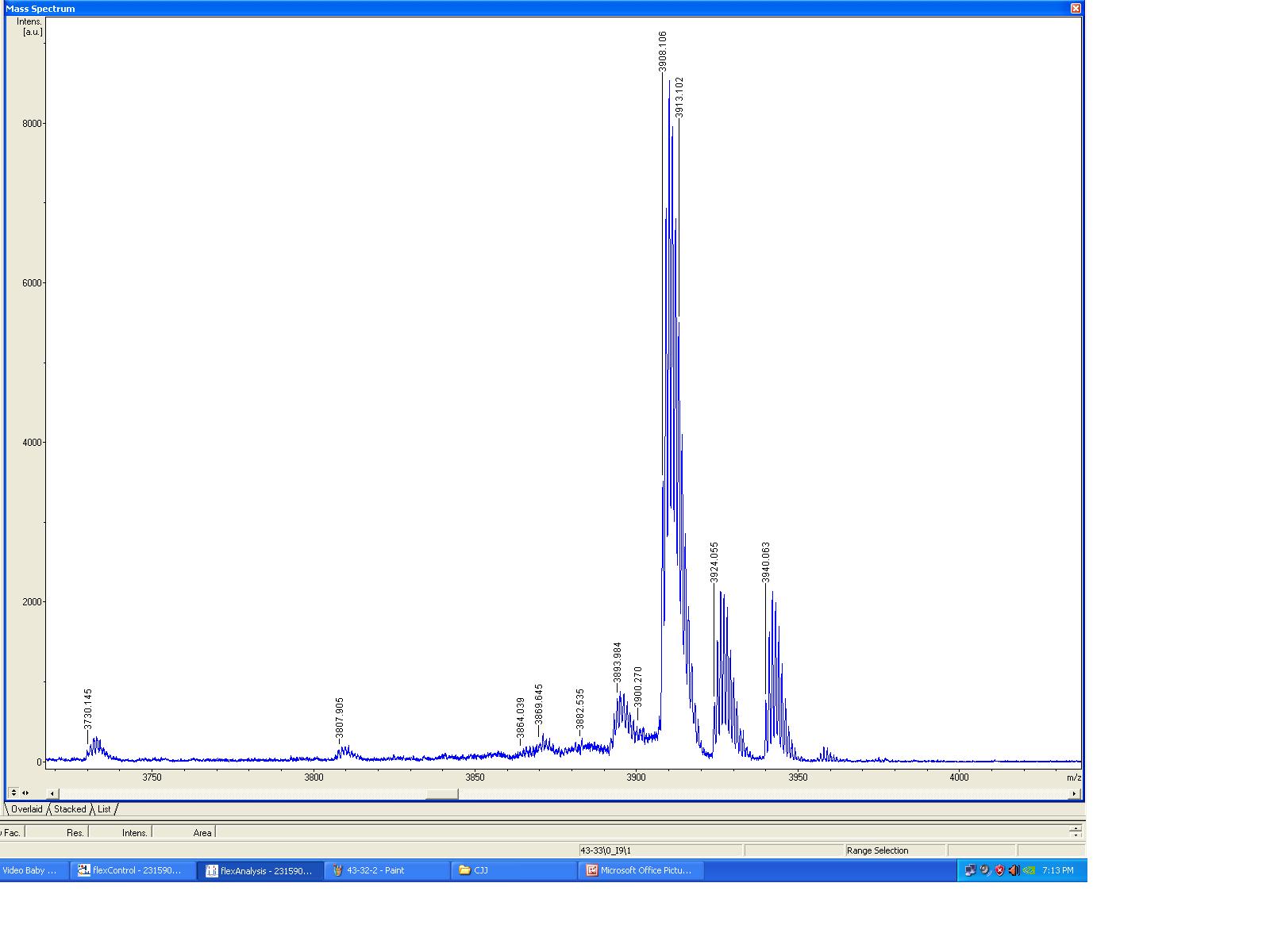


3927


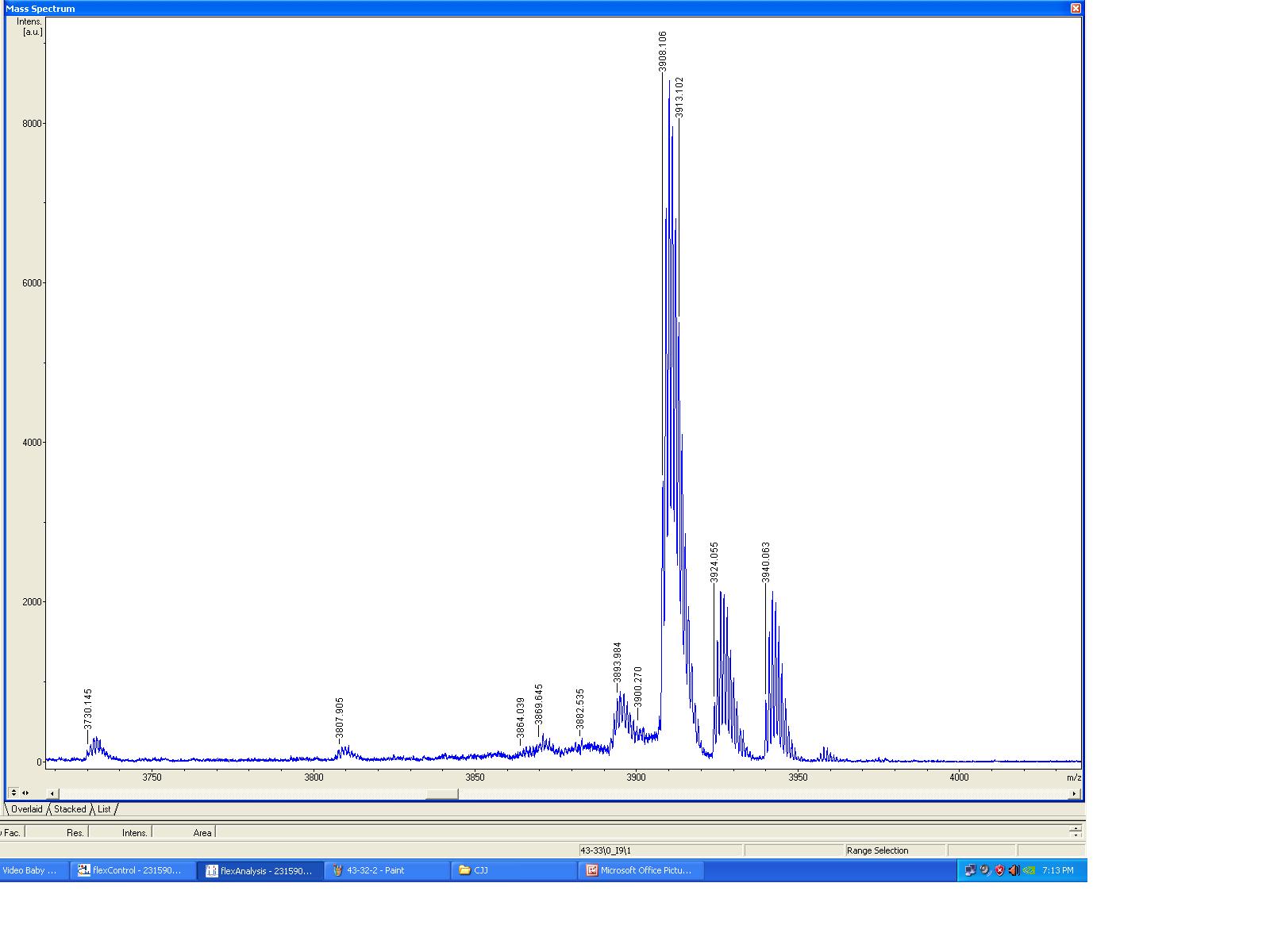


3894


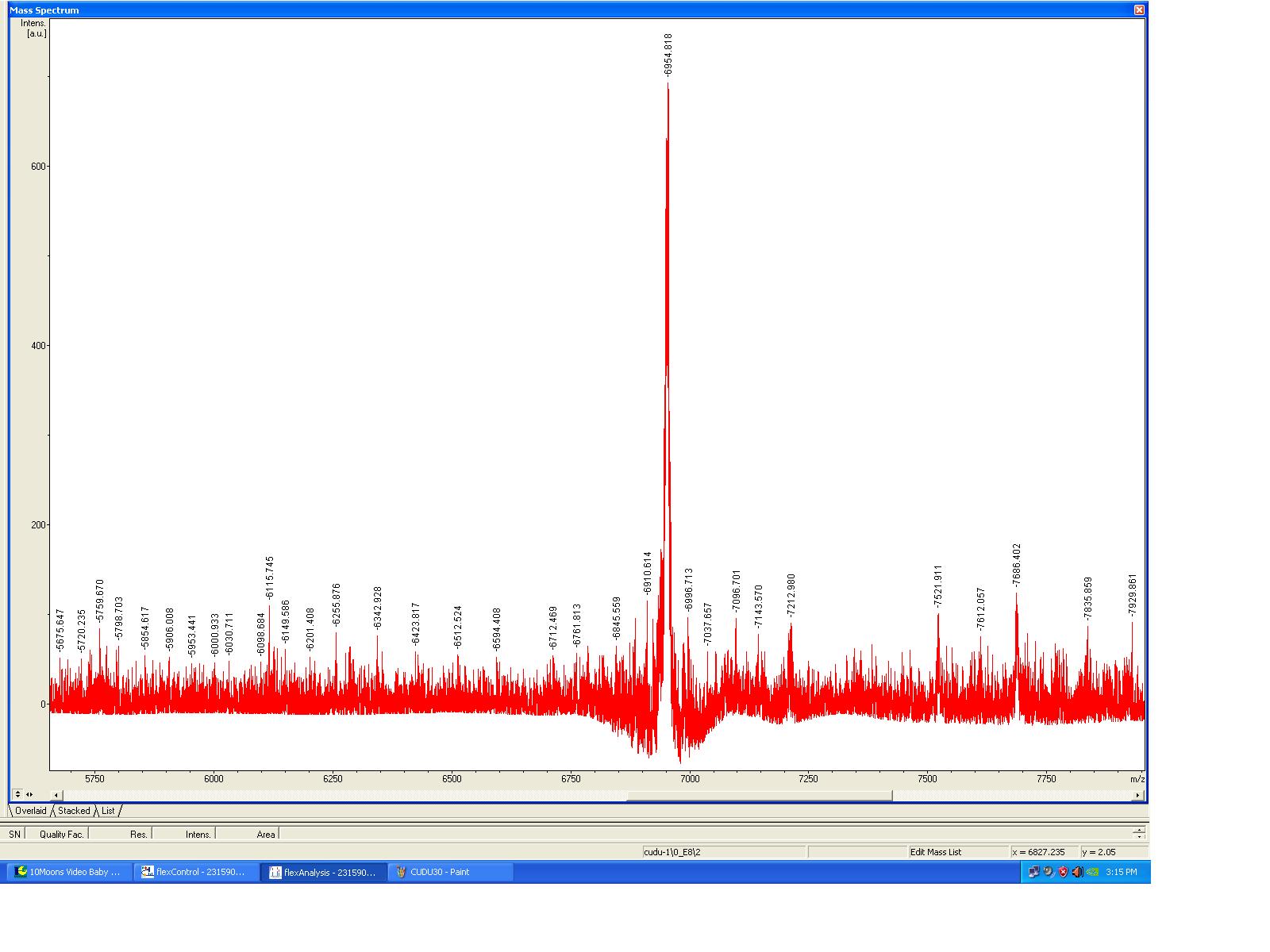


6712


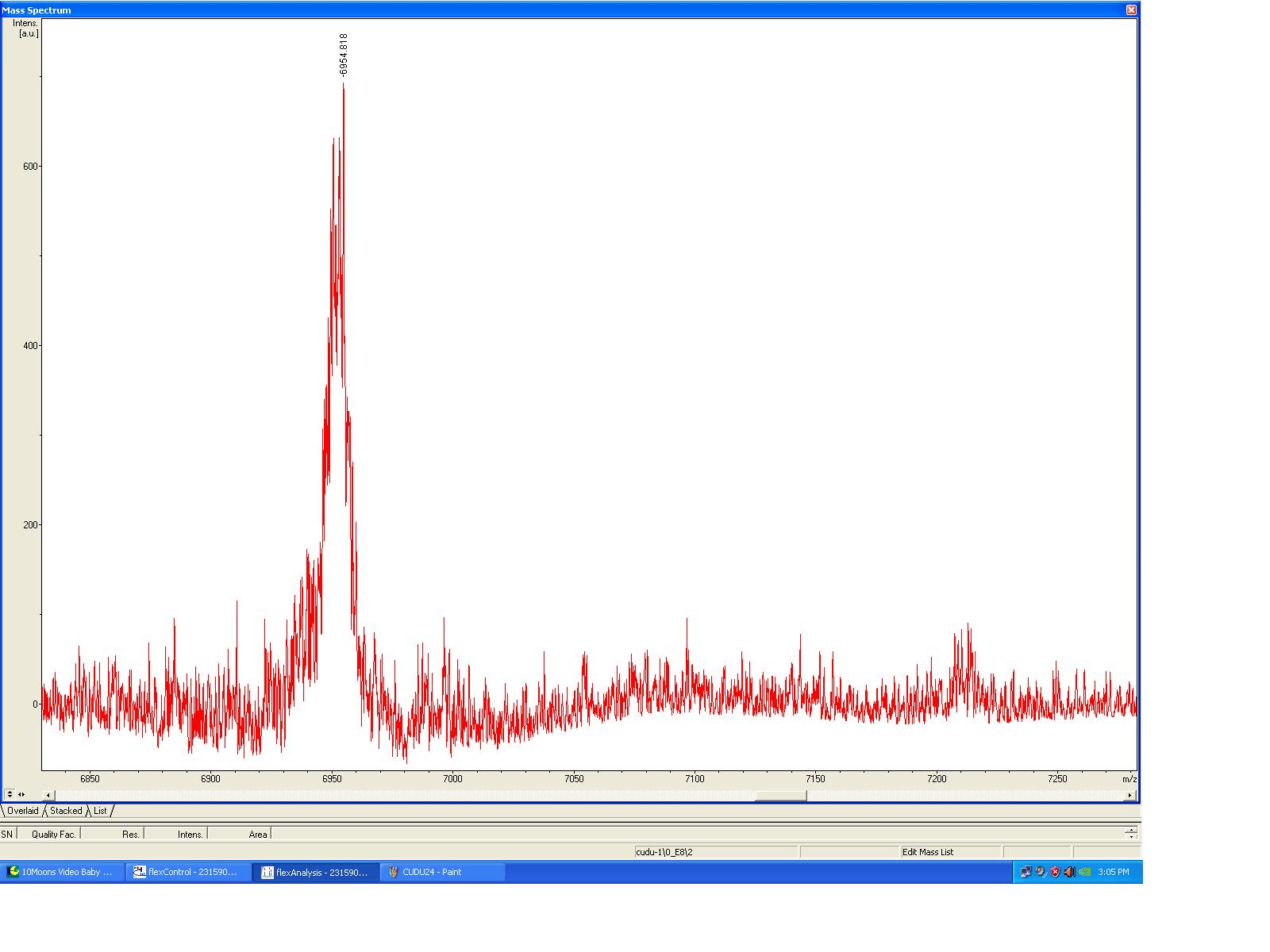


6954


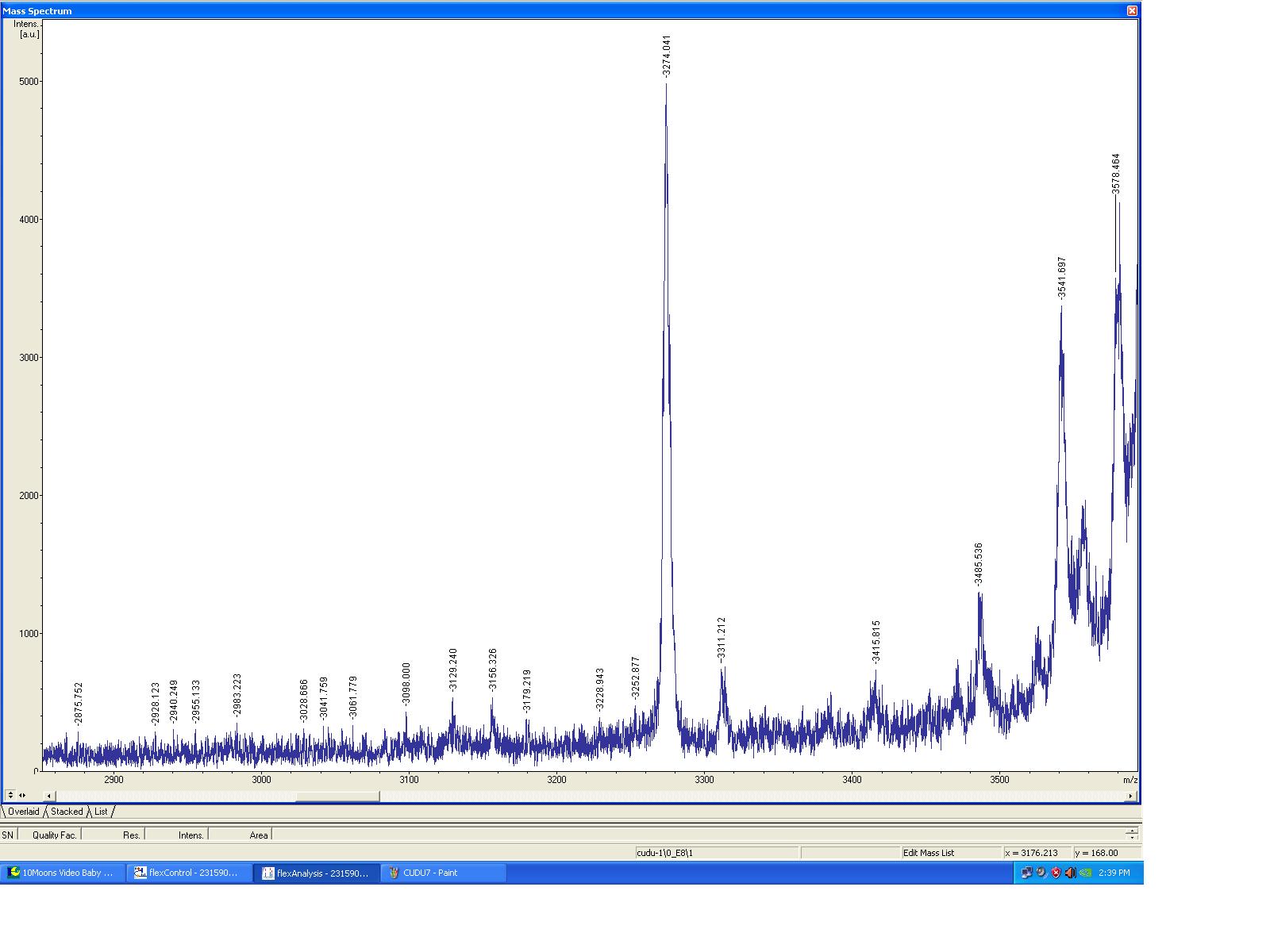


3541


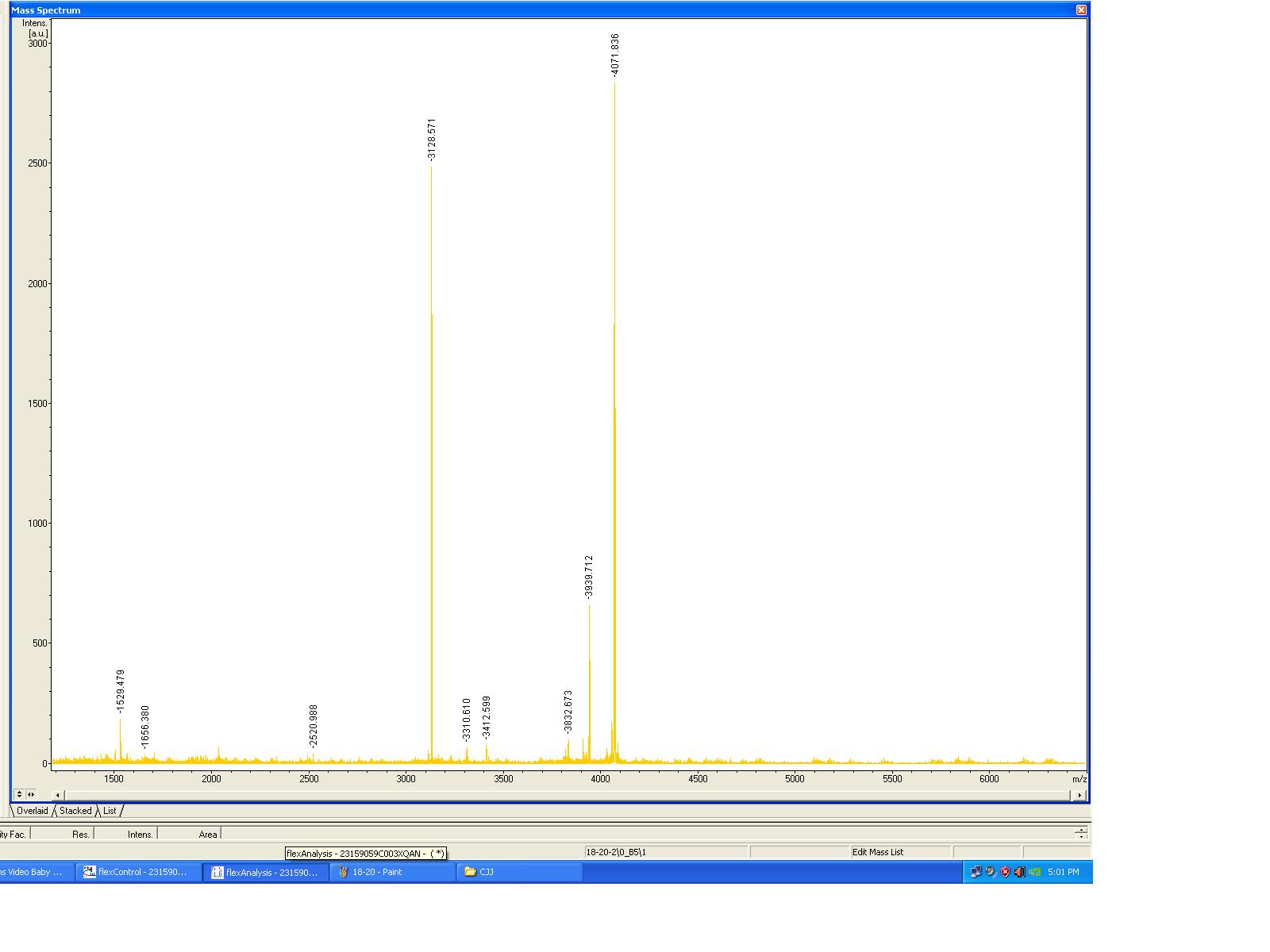


3128
